# Lack of behavioural responses of humpback whales (*Megaptera novaeangliae*) indicate limited effectiveness of sonar mitigation

**DOI:** 10.1242/jeb.161232

**Published:** 2017-11-15

**Authors:** Paul J. Wensveen, Petter H. Kvadsheim, Frans-Peter A. Lam, Alexander M. von Benda-Beckmann, Lise D. Sivle, Fleur Visser, Charlotte Curé, Peter L. Tyack, Patrick J. O. Miller

**Affiliations:** 1 Sea Mammal Research Unit, University of St Andrews, St Andrews, Fife KY16 9TS, UK; 2 Faculty of Life and Environmental Sciences, University of Iceland, Sturlugata 7, 101 Reykjavík, Iceland; 3 Maritime Systems, Norwegian Defence Research Establishment (FFI), NO-3191, Horten, Norway; 4 Acoustics and Sonar, The Netherlands Organisation for Applied Scientific Research (TNO), PO Box 96864, The Hague, 2509 JG, The Netherlands; 5 Marine Ecosystem Acoustics, Institute of Marine Research, PO Box 1870, Nordnes, 5817 Bergen, Norway; 6 Kelp Marine Research, Loniusstraat 9, 1624 CJ, Hoorn, The Netherlands; 7 Behavioural Biology Group, Institute of Biology, Leiden University, PO Box 9505, 2300 RA, Leiden, The Netherlands; 8 Acoustics Group, CEREMA - DTerEst, F-67035, Strasbourg Cedex 2, France

**Keywords:** Behavioural effects, Hearing loss, Naval sonar, Baleen whale, Anthropogenic noise, Ramp-up

## Abstract

Exposure to underwater sound can cause permanent hearing loss and other physiological effects in marine animals. To reduce this risk, naval sonars are sometimes gradually increased in intensity at the start of transmission (‘ramp-up’). Here, we conducted experiments in which tagged humpback whales were approached with a ship to test whether a sonar operation preceded by ramp-up reduced three risk indicators – maximum sound pressure level (SPL_max_), cumulative sound exposure level (SEL_cum_) and minimum source–whale range (*R*_min_) – compared with a sonar operation not preceded by ramp-up. Whales were subject to one no-sonar control session and either two successive ramp-up sessions (RampUp1, RampUp2) or a ramp-up session (RampUp1) and a full-power session (FullPower). Full-power sessions were conducted only twice; for other whales we used acoustic modelling that assumed transmission of the full-power sequence during their no-sonar control. Averaged over all whales, risk indicators in RampUp1 (*n*=11) differed significantly from those in FullPower (*n*=12) by −3.0 dB (SPL_max_), −2.0 dB (SEL_cum_) and +168 m (*R*_min_), but not significantly from those in RampUp2 (*n*=9). Only five whales in RampUp1, four whales in RampUp2 and none in FullPower or control sessions avoided the sound source. For RampUp1, we found statistically significant differences in risk indicators between whales that avoided the sonar and whales that did not: −4.7 dB (SPL_max_), −3.4 dB (SEL_cum_) and +291 m (*R*_min_). In contrast, for RampUp2, these differences were smaller and not significant. This study suggests that sonar ramp-up has a positive but limited mitigative effect for humpback whales overall, but that ramp-up can reduce the risk of harm more effectively in situations when animals are more responsive and likely to avoid the sonar, e.g. owing to novelty of the stimulus, when they are in the path of an approaching sonar ship.

## Abstract (precis)

**Highlighted Article:** Variation in behavioural responses of humpback whales limits the effectiveness of a ramp-up procedure used to mitigate physiological effects of naval sonar on marine animals.

## INTRODUCTION

Noise-induced hearing loss may affect individual survival, as acoustic communication in marine animals facilitates vital behaviours such as feeding and resting, and decisions about habitat selection via social information (Laiolo, 2010). Guidelines for human activities at sea that generate high-intensity sound currently recommend the use of operational mitigation measures designed to protect marine animals. One such mitigation measure is the gradual increase of source intensity prior to normal (full-power) operation, known as ‘ramp-up’ or ‘soft-start’. Ramp-up is used by several navies during sonar exercises (Dolman et al., 2009) and is also common for other activities that involve high-intensity sources of sound, e.g. seismic surveys for oil and gas exploration (Nelms et al., 2016; Dunlop et al., 2016), and pile driving and detonation of explosions during offshore construction (Jefferson et al., 2009). The rationale behind ramp-up varies across sound producers, but the general argument is that for animals that would be close to the source on start-up, ramp-up prior to the start gives these animals time to move away before full-power transmission is reached. Ramp-up procedures for sonar operations are intended to mitigate against permanent hearing loss and other types of physiological effects in animals that are relatively close to the source, but might also protect against severe forms of behavioural disturbance (e.g. panic) in animals near a source that starts at full intensity.

Testing the effectiveness of ramp-up and other noise mitigation measures has been highlighted as a high priority for research in the scientific, conservation and regulatory communities (Southall et al., 2009; Shannon et al., 2016), indicating a clear societal need for such evaluations. Some operational mitigation procedures may prove expensive or affect the fidelity of naval combat training, making it unlikely that producers of underwater noise will adopt such procedures without specific regulations based upon empirical evidence of their conservation benefit. Already in 2009, a NATO working group on marine mammal risk mitigation identified testing the mitigation efficiency of operational methods such as ramp-up and visual-and-acoustic monitoring as a key data gap (R. Dekeling, personal communication). Thus far, however, the effectiveness of ramp-up for naval sonar has yet to be experimentally tested.

Using theoretical modelling, von Benda-Beckmann et al. (2014) found that ramp-up before normal sonar operation can be effective at reducing the number of simulated animals exposed to sound doses that are assumed to be high enough to cause temporary or permanent hearing loss. Important factors are the modelled relationship between acoustic dose and speed of the animals' avoidance response, as well as the ramp-up duration, sailing speed and time interval between the sonar pulses (von Benda-Beckmann et al., 2014). However, even though that study used relevant empirical input in their model, experimental confirmation of these predictions is still missing. It is therefore important to test the effectiveness of ramp-up in realistic conditions at sea.

List of symbols and abbreviations

*a*: gamma shape
*b*: gamma scale
*E*: single-pulse sound exposure
GEE: generalized estimating equation
PL: propagation loss
PTS: permanent hearing threshold shift
*r*: Pearson's correlation coefficient
*R*_min_: minimum source–whale range
SEL: single-pulse sound exposure level
SEL_cum_: cumulative sound exposure level of the session
SL: source level based on mean-square sound pressure
SL_E_: energy source level
SPL: sound pressure level
SPL_max_: maximum sound pressure level of the session
*T*: effective pulse duration
*t*_0_: reference time=1 s
TTS: temporary hearing threshold shift
*Z*: test statistic for Wald test and Barnard's unconditional test
κ: concentration
µ: mean
σ: standard deviation

In this study, the objective was to experimentally test the effectiveness of ramp-up of sonar transmitted by a moving ship on a cosmopolitan cetacean species, the humpback whale (*Megaptera novaeangliae* Borowski 1781). After being exploited during the whaling era, most humpback whale populations have been recovering relatively quickly compared with several other baleen whale sub-populations, many of which remain endangered or critically endangered (Thomas et al., 2016). The distribution of humpback whales during fitness-enhancing behaviours such as feeding and mating is concentrated at inshore and continental shelf waters (Christensen et al., 1992; Rosenbaum et al., 2014), which can overlap with that of naval sonar activity.

In the last two decades, several navies have started using lower-frequency (≤2 kHz) active sonar systems for longer-range detections of submarines (Scott, 2015). These towed systems can ensonify larger areas of ocean as their signals are less directional and less influenced by absorption than higher-frequency signals, and thus have greater potential to affect humpback whales behaviourally or physiologically and to mask their acoustic communication.

Effectiveness of ramp-up of 1.3–2.0 kHz sonar was assessed in terms of whether risk of harm was less in whales presented with a full-power sequence preceded by a ramp-up sequence, i.e. the RampUp session, compared with whales presented with a full-power sequence preceded by a period without sonar, i.e. the FullPower session. In addition, the potential for short-term habituation or sensitization to the sonar exposure was investigated by presenting whales with two identical RampUp sessions. We analysed three risk indicators: maximum sound pressure level (SPL_max_), cumulative sound exposure level (SEL_cum_) and minimum source–whale range (*R*_min_). In addition, effectiveness of ramp-up was assessed by identifying avoidance responses to the sound source and determining their influence on the risk indicators. We tested the specific scenario in which a whale was located directly in front of an approaching source ship and the sonar operation commenced relatively close to the whale, as that scenario likely poses the greatest risk of harm to whales in their natural environment.

## MATERIALS AND METHODS

### Data collection and experimental protocol

Fieldwork was conducted in the Barents Sea between Bear Island and Spitsbergen in June 2011 and 2012 aboard the 55-m research vessel H. U. Sverdrup II (Kvadsheim et al., 2011). Details of the experimental protocols are described elsewhere (Kvadsheim et al., 2015) and summarized here. Humpback whales were detected visually from the flying bridge of the research vessel. After a whale was sighted, a tag boat was launched to deploy a multi-sensor tag [DTAG; Woods Hole Oceanographic Institution (WHOI), Woods Hole, MA, USA; Johnson and Tyack, 2003]. Tags were attached to the whale with suction cups using a cantilevered carbon fibre pole, or a pneumatic remote deployment system. DTAGs recorded sound (96 kHz; 16 bits), pressure, three-axial acceleration and three-axial magnetic field strength (50 Hz each). DTAGs also included a VHF beacon to aid visual tracking, and carried a small Fastloc-GPS logger (F2G 134A, Sirtrack, Havelock North, New Zealand) to record GPS surface locations. After a tagging period, groups of one or two tagged whales were tracked from an 8-m motorized boat at ∼100–200 m. Observers recorded the distance and bearing to the focal whale, and number of whales in the group, for each new surfacing occurring at least 2 min after the last record. Pairs of whales were considered potential mother–calf pairs if they were composed of an adult and a smaller-sized individual that remained closely associated throughout the tracking record (Curé et al., 2015).

Experimental sessions started after 4 h (2012) or 8 h (2011) of baseline data were collected per whale. Each tagged whale was subject to three experimental sessions: first a no-sonar control, then two sonar sessions. Sessions had a 10-min duration each and were separated by 1 h or more. The no-sonar control was always conducted first to test how whales responded to the ship alone, before they heard sonar transmitted from the vessel. During no-sonar control the ship approached the tagged whale in the same way as during sonar sessions, but without transmitting sonar.

**Fig. 1.**
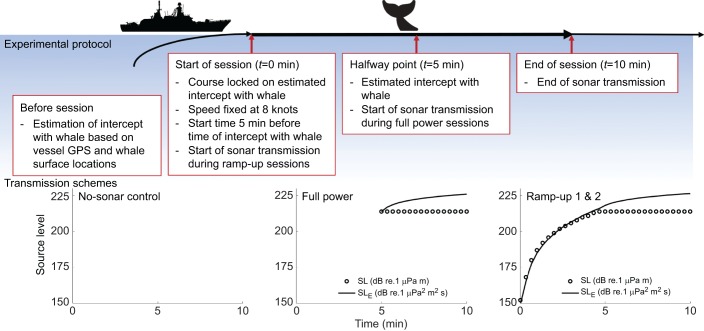
**The experimental protocol, including navigational protocol of the source ship and transmission schemes of the sonar.** Session names reflect both the stimulus presentation (no-sonar control, full-power and ramp-up) and order of presentation (ramp-up 1 and ramp-up 2). The navigational protocol was identical across experimental sessions but which transmission scheme was used depended on the type of session. The bottom panels show the source level in terms of RMS sound pressure (dotted line; dB re. 1 μPa m) as well as cumulative sound exposure (dB re. 1 μPa^2^ m^2^ s).

The source ship approached the whale at a speed of 4.1 m s^−1^ (8 knots) and on a predetermined straight intercept course during no-sonar control and sonar sessions (see Fig. 1 for a schematic of the navigational protocol). Both the line-of-approach and start time of the experimental session depended upon a prediction of the future track of the whale: each session started at 1.25 km from an estimated intercept with the tagged whale, as this was the distance covered during 5 min at 4.1 m s^−1^ sailing speed. Each session was conducted using two independent intercept calculator tools to support navigation during the vessel approach: MARIA GDK (Teleplan Globe, Lysaker, Norway), developed for the Norwegian Navy, and RU-tool (custom-built in MATLAB by P.J.W.). The surface positions of the whale, relayed via VHF radio by the observers on the tracking boat, and the GPS positions of the source ship were fed into the tools, which then calculated the time and position of the intercept and the best sailing path. The two tools used somewhat different logic: MARIA primarily used the automatic identification system signal from the tracking boat, which was usually around 100 m from the whale; and RU-tool predicted the future movements of the whale from the last sightings by applying different rules based on the whale's general movement pattern. The final course correction and start time of the session were determined only minutes before the start. Ultimate decisions on course changes and start of transmission were always made by the experiment coordinator based on all available information.

The two sonar sessions were either two identical ramp-up sessions (‘RampUp1’ and ‘RampUp2’) or a ramp-up session (‘RampUp1’) and a full-power session (‘FullPower’) without ramp-up (Table 1). Ramp-up sessions consisted of a 5-min full-power operation preceded by a 5-min ramp-up, and full-power sessions consisted of a 5-min full-power operation preceded by 5 min without sonar transmissions. This design allowed us to test two distinct hypotheses: (1) that sonar operations preceded by ramp-up may be more effective at reducing risk of harm compared with sonar operations that are not preceded by ramp-up, which was tested using the full-power session and first ramp-up session, and (2) that whales may habituate or become sensitised to the sonar signal, which was tested using the two successive ramp-up sessions. The reader is referred to ‘Discussion, Methodological considerations’ for a description of how this design was established.

**Table 1.**
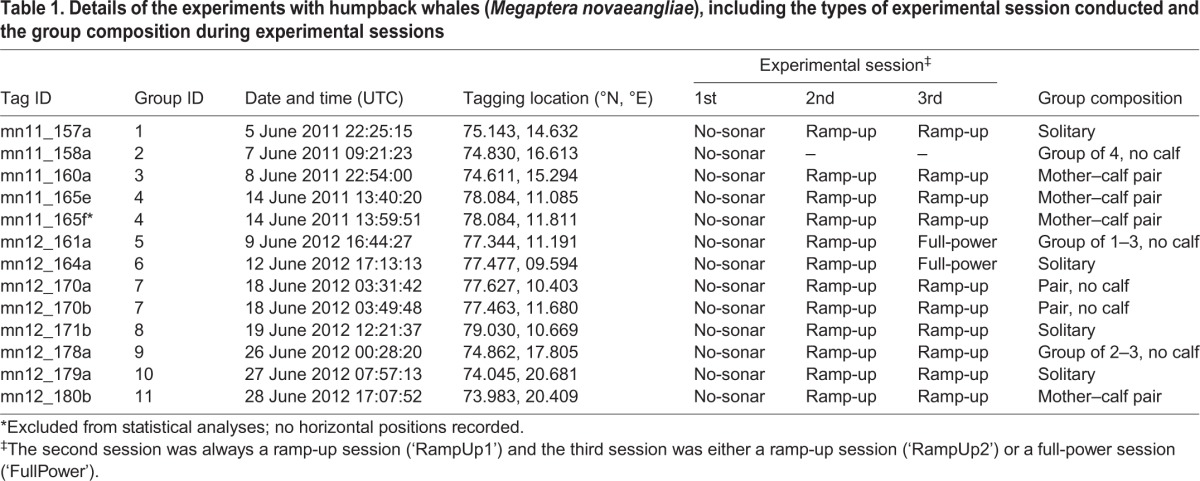
**Details of the experiments with humpback whales (*Megaptera novaeangliae*), including the types of experimental session conducted and the group composition during experimental sessions**

The research vessel towed the sonar source (Socrates II, TNO, The Hague, The Netherlands) at 50 m depth using 250–300 m of cable. The source transmitted a 1.3–2.0 kHz upsweep pulse at 20 s interval. The single-pulse source level (SL; ISO18405:2017) was gradually increased from 152 to 214 dB re. 1 µPa m in the first 5 min and then kept at 214 dB re. 1 µPa m during ramp-up sessions (Fig. 1). Full-power sessions had no sonar transmission in the first 5 min and SL=214 dB re. 1 µPa m in the last 5 min (Fig. 1). The pulse duration was 1 s, except for the first 5 min of the ramp-up session, in which it was 0.5 s. We shortened the pulse duration to 0.5 s with the premise that the resulting reduction in cumulative exposure should reduce risk of hearing loss (von Benda-Beckmann et al., 2014).

At present, different ramp-up schemes are in operation worldwide. We could only test one ramp-up experimentally; therefore, the ramp-up scheme was based upon considerations of operational relevance and a quantitative assessment of risk of hearing loss. In this quantitative assessment, we applied a model framework described elsewhere (von Benda-Beckmann et al., 2014) with parameter values adjusted for humpback whales (speed: 1.0 m s^−1^) and sonar source used (pulse length during ramp-up phase: 0.2 s; other parameters as described above; details in Kvadsheim et al., 2011). The final parameter values for ramp-up duration, SL increase and range, and pulse duration, shape and interval, used during the at-sea experiments, were assumed to maximize the probability of avoidance and minimize the risk of hearing loss.

The animal experiments reported here were carried out under permits issued by the Norwegian Animal Research Authority (permit no. S2011/38782), in compliance with the ethical use of animals in experimentation. The research protocol was approved by the University of St Andrews Animal Welfare and Ethics Committee and WHOI's Institutional Animal Care and Use Committee.

### Modelling of full-power outcomes

Full-power sessions were only conducted twice (whales mn12_161a and mn12_164a) and always as the third session (Table 1). Records in the FullPower dataset for 10 other whales were based on (1) modelling of sound exposures that would have been received by the whales if the source had been transmitting the full-power sequence during no-sonar control sessions and (2) observed source–whale proximities during these no-sonar control sessions. The behaviour of the whales was not altered when full-power outcomes were generated. Thus, the FullPower dataset consisted of two whales (two sessions) that were exposed to a full-power sequence and 10 whales (those with horizontal positions recorded; nine sessions) for which the no-sonar control was used. This approach of generating full-power outcomes was taken because the navigational protocol, with source–whale intercept at *t*=5 min, in combination with the full-power transmission sequence, with first the sonar transmission at *t*=5 min (Fig. 1), was determined to not give whales enough time to substantially affect the risk indicators by avoiding the sonar. We simulated a full-power session to statistically assess the effect that an avoidance response to the first sonar transmission could have on SPL_max_, if it occurred (Appendix A). In this simulation, avoidance behavior was modelled by randomly drawing samples from empirical distributions (e.g. for avoidance speed, depth, accuracy of intercept) derived from all observations collected during experimental sessions. The simulation showed that there was a negligible chance that an avoidance response would substantially reduce SPL_max_ (Appendix A).

Use of full-power outcomes in this way was considered the most appropriate approach from an animal-welfare perspective because it (1) reduced the number of times the source suddenly was switched on at maximum level at relatively close range from the tagged animal and (2) allowed us to obtain information about habituation and sensitization by exposing animals to two identical subsequent ramp-up sessions instead of to one ramp-up session and one full-power session. The two experimental full-power sessions were conducted to provide information about potential effects of the sudden nearby onset of sonar on the magnitude of the behavioural response (see ‘Discussion, Methodological considerations’ for the rationale behind this approach).

### Data analysis

We analyzed all experimental sessions to quantify three risk indicators – maximum sound pressure level (SPL_max_), cumulative sound exposure level (SEL_cum_) and minimum source–whale range (*R*_min_) – which we could estimate from our at-sea treatments. Single-pulse sound pressure level (SPL) and sound exposure level (SEL) for sonar transmissions received on the DTAG were measured following the procedures in Miller et al. (2012). For definitions of SPL, SEL and SL we followed ISO 18405:2017 (https://www.iso.org/standard/62406.html). SEL_cum_ is the logarithm of the sum of the single-pulse sound exposures over all pulses in the session, and SPL_max_ is the maximum SPL of all pulses in the session. We combined SL with modelled acoustic propagation loss (PL) using ray-trace software (BELLHOP; Porter and Bucker, 1987) to obtain the received levels, i.e. the SPL and SEL of the sonar exposures received at the location of the whale, for all sonar sessions (Wensveen et al., 2015a). Sound speed profiles used for acoustic modelling were collected on-site using a conductivity-temperature-depth (CTD) profiler or an expendable bathythermograph, either during the experimental session or shortly after the session had ended (Kvadsheim et al., 2011). To correct for systematic discrepancy in the received levels (Fig. S1; Appendix B; see also Sivle et al., 2015; Wensveen et al., 2015a), we used the mean difference between the modelled received levels and the received levels measured on the DTAGs. The slant range between the three-dimensional position of the source and the three-dimensional position of the whale was calculated using high-resolution tracks of the whales, which were estimated from visual, GPS and tag data by Wensveen et al. (2015b). We applied a Monte Carlo simulation to propagate forward uncertainty in the whale's position into the modelled received levels. Further details on the received level analysis are provided in Appendix B.

**Fig. 2.**
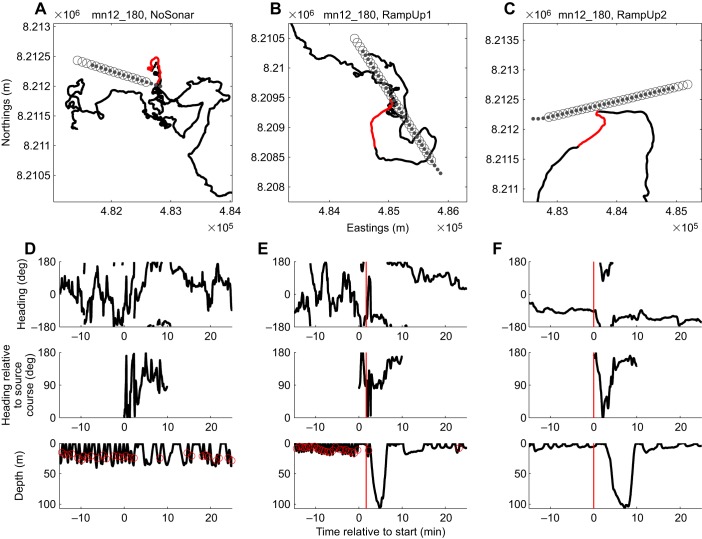
**Movements of a humpback whale mother–calf pair during a controlled exposure experiment.** Top panels show maps with the locations of tagged whale mn12_180b (the mother) and locations of the sonar source during three experimental vessel approaches indicated: (A) no-sonar control session, (B) first ramp-up session and (C) second ramp-up session. The horizontal track of the whale (black line) is shown with the section corresponding to the experimental session highlighted (red line). The location of the sonar source (grey dots) and the location of the research vessel towing the source (grey circles) are shown for each individual sonar transmission. (D–F) Time-series data for the same experimental sessions, plotted as function of time relative to the start of the session: (1) whale heading relative to north, (2) whale heading relative to the course of the ship (where 0 deg represents whale movement in the same direction and 180 deg in the opposite direction) and (3) depth of the whale (black line) with indications when feeding events occurred (red circles). The onset of avoidance during the two ramp-up sessions is marked in the time-series plots (red vertical line). Similar plots were made for all experiments (Fig. S1).

For each session, a quantitative analysis to identify avoidance responses in the whale's horizontal track was conducted. This analysis aimed to replicate the definition of avoidance used by Sivle et al. (2015), who used expert identification of responses based on lower resolution tracks (i.e. only surface positions) for the same whales. Each track was downsampled to time steps of 20 s (the pulse interval) and then used to calculate whale heading. We identified all turns in the tracks and which of these could indicate avoidance. A turn was defined as a track segment in which the change in heading was either positive or negative and composed of one or several time steps. A turn was considered a potential avoidance response if it was ≥90 deg, occurred during a session and was followed by persistent movement away from the ship's trackline (defined as the whale moving at an average absolute heading between 0 and 100 deg relative to the course of the ship; Fig. 2, Fig. S2). To avoid misinterpretation of normal and coincidental changes in behaviour, the turn angle of each potential avoidance was compared with the empirical cumulative distribution of turn angles for all clockwise or counter-clockwise turns during 4 h of baseline data preceding the session. This baseline data set included turns during the period before the no-sonar control and, for sonar sessions, periods between experimental sessions. The duration of 4 h was chosen to reduce differences in the amount of data available per whale and variation in turn angles owing to whales switching behavioural state. Only turns in one direction were used to reduce autocorrelation in the baseline data set. Turns with a *P*-value of less than a Bonferroni-corrected significance level (0.05 divided by the number of tests on the experiment) were classified as avoidance.

We also assigned presence/absence of feeding just before or during the experimental session based upon the detection of lunges (Fig. 1D–F), i.e. feeding events in which the whale speeds up, engulfs a large volume of water and uses its baleen to filter prey (Goldbogen et al., 2008). ‘Feeding’ was assigned if at least one lunge was present in the time interval from 10 min before the start of the session until the onset of the response, or the end of the session if no response was scored. The feeding lunges used were identified by Sivle et al. (2016) at depths >0.5 m using a reliable detection algorithm (Simon et al., 2012).

### Statistical procedures

We compared the presence/absence of avoidance between no-sonar control sessions and ramp-up sessions (RampUp1 and RampUp2 combined) using a two-sided Barnard's unconditional test of superiority on a contingency table with avoidance (no response, response) and a null hypothesis on no effect of ramp-up session, relative to no-sonar control.

We were interested in examining whether the risk indicators (SPL_max_, SEL_cum_ and *R*_min_) changed across the three types of sonar session and across whales that avoided the source versus whales that did not. We used generalized estimating equations (GEEs), which are an extension of generalized linear models that accounts for correlated and clustered data by appropriately inflating the standard errors (Hardin and Hilbe, 2003). The dependent variable in the models was the risk indicator. SPL_max_ and SEL_cum_ were modelled using Gaussian distributions and *R*_min_ using a gamma distribution.

All explanatory variables were factor covariates. We fitted models with session (FullPower, RampUp1, RampUp2) as the explanatory variable to examine the effects of transmission scheme and presentation order. As described above, we pooled 10 full-power outcomes that were based on no-sonar control sessions with two observed full-power sessions to create the FullPower dataset. This pooling assumed that the behaviour of the two whales during full-power sessions was unaffected by the previous two sessions. This assumption appears to be reasonable based on the movement tracks of the two exposed animals (Fig. S2E,F). All tagged whales were used in the analysis, resembling the real-world situation where animals encountering a sonar ship may or may not exhibit avoidance. We also fitted models with avoidance, ramp-up session (RampUp1, RampUp2) and their interaction as explanatory variables to examine the effects of avoidance and presentation order. Only ramp-up sessions were used in this analysis because avoidance responses were not observed in full-power sessions.

Group ID was selected as blocking unit because data from both mn12_170a and mn12_170b were included and the behaviour of these associated whales may be not independent. All exposed whales were included in the statistical analysis except for whale mn11_165f, as visual or GPS fixes were not recorded for this (non-focal) animal. The jackknife variance estimator was applied because the sandwich variance estimator can be biased for small sample sizes (Højsgaard et al., 2014). An independent correlation structure was used in all GEE analyses. We verified that three competing working correlation structures, i.e. exchangeable, AR1 and unstructured, did not improve the quasi-likelihood under the independence model (Hardin and Hilbe, 2003).

For inference purposes, we used prediction plots generated from the GEE models. The 95% confidence intervals (CIs) for the predictions were calculated using a parametric bootstrap on the GEE covariance matrix. The 2.5th and 97.5th percentiles were calculated from 5000 bootstrap iterations. We used two-sided Wald tests to assess differences between factor levels in models with session. For models with avoidance and ramp-up session, we used the output from the 5000 bootstraps to make comparisons between specific factor level combinations of interest, as the interaction term precluded the use of the standard Walt tests. We calculated the differences between predictions for different factor levels across all bootstraps as well as a 95% CI for these differences. We concluded that there was a significant increase or decrease in the risk indicator in cases where the upper and lower confidence bounds for the differences were exclusively positive or negative.

All statistical analyses were performed in R version 3.0.2 using R packages geepack (Højsgaard et al., 2014), MuMIn (Barton, 2015) and Barnard (Erguler, 2015). The raw data are available as Table S1.

## RESULTS

We successfully tagged 13 humpback whales, five in 2011 and eight in 2012 (Table 1). One whale was subject only to the no-sonar control because of a premature tag release. The remaining 12 whales were subject to three experimental sessions: first a no-sonar control, then two sonar sessions. These sonar sessions were either two consecutive ramp-up sessions (10 whales) or a ramp-up session followed by a full-power session (two whales; Table 1). The 13 whales were part of 11 independent groups of one or two tagged whales; on two occasions (mn11_165ef and mn12_170ab), two whales in the same group were tagged (Table 1). The number of groups tagged and exposed to sonar was based on a power analysis conducted after the first field season.

Whales were exposed to mean SPL_max_=173 dB re. 1 µPa (s.d. 4 dB) and SEL_cum_=177 dB re. 1 µPa^2^ s (s.d. 3 dB) over all ramp-up and full-power sessions. The source passed at relatively close range to the whale: mean *R*_min_ was 299 m (s.d. 209 m) over all sessions. The experimental design, with the whale in front of an approaching source, resulted in strong correlations among the three risk indicators: SPL_max_ and SEL_cum_ (*r*=+0.92, *P*<0.001), SPL_max_ and *R*_min_ (*r*=−0.93, *P*<0.001), and SEL_cum_ and *R*_min_ (*r*=−0.84, *P*<0.001).

Avoidance responses were identified in eight ramp-up sessions (nine whales) but never in the 11 no-sonar control sessions or two full-power sessions (Fig. S2). The presence of avoidance thus differed between stimulus presentations (Barnard's test: *Z*=2.8, *P*=0.005), suggesting that responses were caused by the sonar exposure and not the approaching ship. Between-whale and within-whale variations in the presence of avoidance were large; avoidance occurred during five of 10 RampUp1 sessions (five of 11 whales) and three of eight RampUp2 sessions (four of nine whales). Only one whale (mn12_180b) avoided the source during both RampUp1 and RampUp2 (Fig. 1B,C). Of whales that avoided, four whales were in a feeding state and five whales were in a non-feeding state prior to the response. All avoidance responses were initiated when the source was approaching the whale (Fig. S2).

We investigated the effectiveness of ramp-up on humpback whales using statistical models with session (FullPower, RampUp1, RampUp2) as explanatory variable. FullPower consisted of observed sessions (two whales) and modelled outcomes (10 whales). The average differences in risk indicators between RampUp1 and FullPower were small but statistically significant (Fig. 2; Table 2). Differences for RampUp1 relative to FullPower were −3.0 dB for SPL_max_ (*Z*=7.6, *P*=0.006), −2.0 dB for SEL_cum_ (*Z*=11.6, *P*<0.001) and +168 m for R_min_ (*Z*=8.8, *P*=0.003; Table 2). None of the contrasts with RampUp2 were statistically significant (Fig. 2, Table 2), and for all three risk indicators, the mean values in RampUp2 were closer to those in FullPower than to those in RampUp1.

**Table 2.**
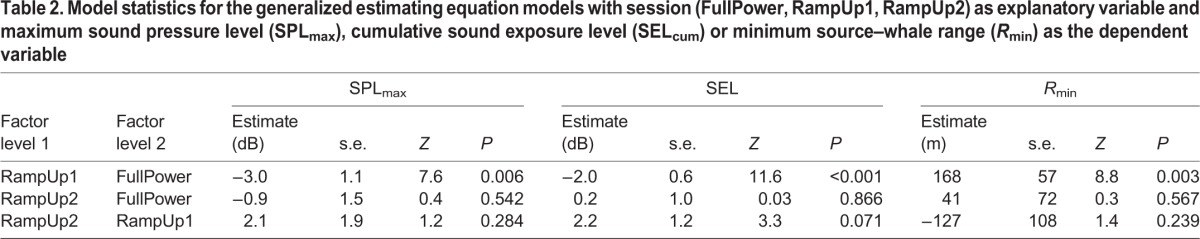
**Model statistics for the generalized estimating equation models with session (FullPower, RampUp1, RampUp2) as explanatory variable and maximum sound pressure level (SPL_max_), cumulative sound exposure level (SEL_cum_) or minimum source–whale range (*R*_min_) as the dependent variable**

Because effectiveness of ramp-up is mediated by the likelihood of inducing avoidance and by characteristics of the response (e.g. swim speed, heading), we also compared whales that avoided the sound source with whales that did not avoid. For RampUp1, we found statistically significant differences between these two categories: −4.7 dB for SPL_max_, −3.4 dB for SEL_cum_ and +291 m for *R*_min_ (Fig. 2, Table 3). In contrast, for RampUp2, these differences were smaller overall and not statistically significant (Fig. 2, Table 3). Whales avoiding in RampUp2 received higher levels overall than whales avoiding in RampUp1 (+3.6 dB for SPL_max_ and +4.1 dB for SEL_cum_) and their minimum distance to the source averaged 209 m lower (Fig. 2, Table 3). These differences between whales that avoided were significant for SEL, but this was not the case for SPL_max_ and *R*_min_ (Table 3). Thus, while approximately the same percentage of whales avoided the sonar during RampUp1 compared with RampUp2, avoidance responses reduced received levels more effectively during RampUp1.

**Table 3.**
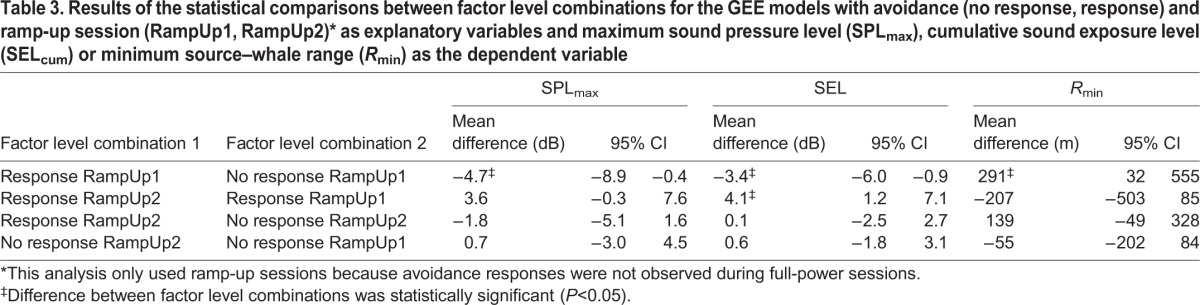
Results of the statistical comparisons between factor level combinations for the GEE models with avoidance (no response, response) and ramp-up session (RampUp1, RampUp2)* as explanatory variables and maximum sound pressure level (SPL_max_), cumulative sound exposure level (SEL_cum_) or minimum source–whale range (*R*_min_) as the dependent variable

There were indications that a combination of behavioural state (feeding, non-feeding) and order of presentation influenced avoidance behaviour. The three whales in RampUp1 that initiated avoidance from a non-feeding state received a mean SPL_max_=167 dB re. 1 µPa (s.d. 5 dB), which was ∼6 dB lower than other whales that avoided [feeding/RampUp1: 173 dB re. 1 µPa (s.d. 2 dB), *n*=2; non-feeding/RampUp2: 174 dB re. 1 µPa (s.d. 2 dB), *n*=2; feeding/RampUp2: 172 dB re. 1 µPa (s.d. 0 dB), *n*=2]. Similar trends were observed for SEL_cum_ and *R*_min_: whales that initiated avoidance from a non-feeding state in RampUp1 generally had a lower SEL_cum_ and a greater *R*_min_ than other whales that avoided.

## DISCUSSION

### Effectiveness of ramp-up for humpback whales

Ramp-up of 1.3–2.0 kHz sonar reduced risk, i.e. a decrease in SPL_max_ and SEL_cum_ and increase in minimum distance to the whale, of exposure to humpback whales, but only in the first sonar exposure session (RampUp1). The average reductions in risk for RampUp1 compared with FullPower were statistically significant, but relatively small (Fig. 2, Table 2). Animals exposed to an identical ramp-up sequence during two consecutive sessions did not exhibit similar risk reductions in the second session. However, unlike the differences in risk found between RampUp1 and FullPower, the differences in risk between RampUp1 and RampUp2 were not statistically significant, probably because of the smaller sample size and greater variation in responsiveness during RampUp2 (Table 2).

**Fig. 3.**
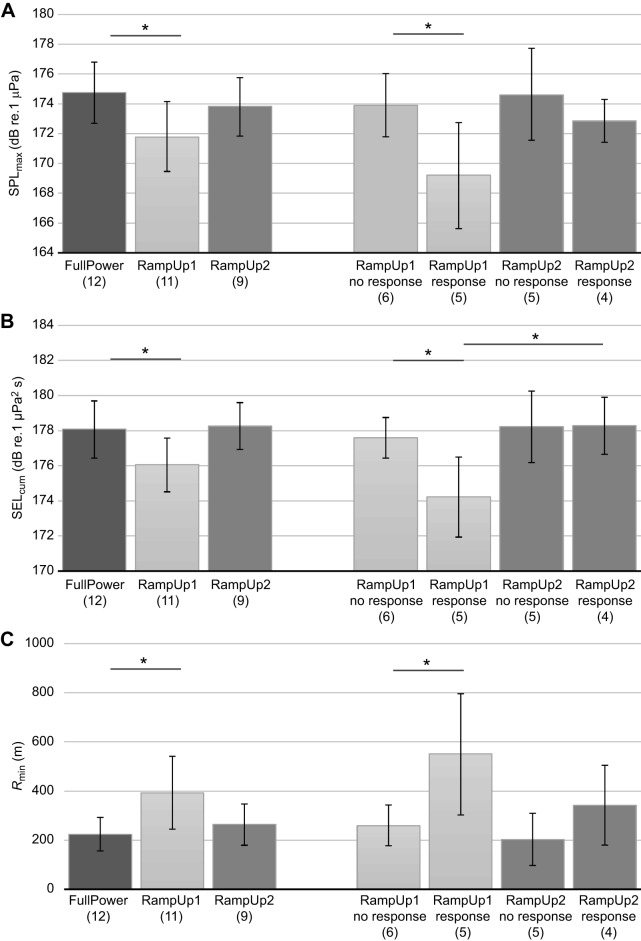
**Generalized estimating equation (GEE) model predictions for the risk indicators measured.** Risk of harm was indicated using (A) maximum sound pressure level (SPL_max_), (B) cumulative sound exposure level (SEL_cum_) or (C) minimum source–whale range (*R*_min_). Significant (*P*<0.05) differences between factor levels or factor level combinations are indicated by an asterisk. Note that combination RampUp1/response was not statistically compared with combination RampUp2/no response (Table 3). Error bars indicate 95% confidence intervals obtained from a parametric bootstrap on the GEE covariance matrix using 5000 iterations. The number of whales (*n*) is shown in parentheses below each bar.

Changes in heading found to be avoidance responses were identified in approximately half of the whales (Fig. 3), which explains the small reductions in risk when averaged over all whales in the session. Whales that avoided the source during RampUp1 significantly reduced risk compared with whales that did not avoid during RampUp1, but also reduced their received level risk indicators compared with whales that avoided the source during RampUp2 (Fig. 2, Table 3). Thus, avoidance behaviour only reduced risk when whales had not been exposed to sonar 1 h before and the efficacy of ramp-up thus appeared to be influenced by the novelty of the stimulus. Such context-specificity is considered important for behavioural responses of humpback whales and other marine mammals (Ellison et al., 2012; Harris et al., in press). Furthermore, avoidance responses by the three whales that were in a non-feeding state during RampUp1 appeared to reduce risk more than avoidance responses by whales that were in a feeding state, suggesting that the behavioural responses of humpback whales to naval sonar varied with behavioural state, as in blue whales (*Balaenoptera musculus*; Goldbogen et al., 2013).

Five of the nine tagged whales exposed to two ramp-up sessions exhibited avoidance in only one session. This could be due to random variation in behavioural responses to man-made noise, common in baleen whales (McCauley et al., 2000; Goldbogen et al., 2013). An alternative explanation is that some of the whales were sensitized by the sonar whilst others habituated. The same humpback whales exhibited much stronger avoidance responses when killer whale sounds were played from a small drifting boat, at lower received levels than the sonar, documenting the whales' ability to respond more strongly, and indicating differences in level of perceived risk between the two stimuli (Curé et al., 2015; Sivle et al., 2015).

We did not analyze behavioural responses other than avoidance, but such responses (e.g. feeding cessations) have been reported in parallel studies of the same experiments (Sivle et al., 2015, 2016). These studies found a general tendency of habituation also in other aspects of the humpbacks' behavioural responses. A presentation-order effect might have occurred owing to the no-sonar control always being presented first. However, only one behavioural response (a brief cessation of feeding; Sivle et al., 2015) was observed during a no-sonar control session that was followed by an avoidance response in RampUp1, and this animal, mn12_180b, did not habituate to the sonar during RampUp2. While a lack of response during the no-sonar control does not mean animals could not be sensitized by it, we consider this unlikely to have affected the overall pattern of habituation to the sonar.

**Fig. 4.**
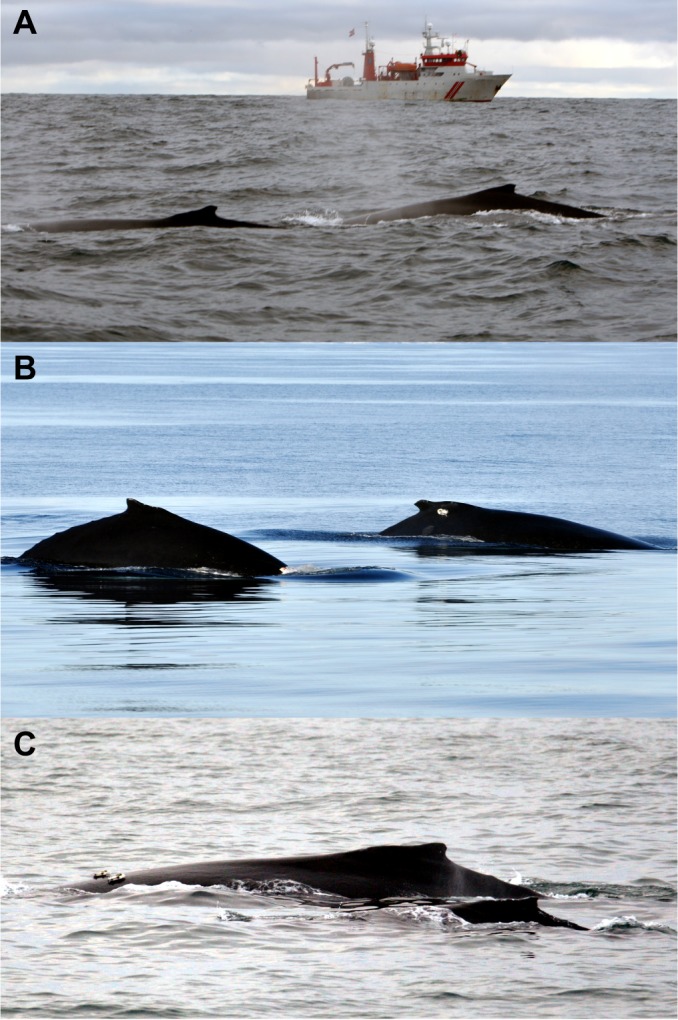
**Photographs of the potential humpback whale mother–calf pairs.** Groups of one or two tagged whales exposed to sonar included three potential mother–calf pairs: (A) mn11_160a and an untagged adult (with the source ship in the background), (B) mn11_165e and calf mn11_165f, and (C) mn12_180b and an untagged calf. Note that the calf of mn12_180b was substantially smaller than the other two calves.

The only tagged whale that avoided during both sonar sessions was a mother (mn12_180b) with a small calf. Whale mn12_180b responded during both ramp-up sessions with an unusual three-dimensional avoidance response, which included a descent to >100 m depth (Fig. 2E,F). The calf of mn12_180b was substantially smaller than the other two calves (Fig. 4). Dive behaviour of the calf was not recorded as we did not tag it; however, this animal was always near its presumed mother when she surfaced. Young humpback whale calves produce very quiet calls while they are swimming (Videsen et al., 2017), so diving deep and increasing the horizontal distance to a masking sound source could help the mother to stay in contact with her calf. The observation is also consistent with the suggestion that mother–calf pairs are more likely to exhibit avoidance responses to man-made sounds that they are unaccustomed to (McCauley et al., 2000). We speculate that the mitigative effect of ramp-up may thus be higher for mothers with young calves compared with other groups. That the other two potential mother–calf pairs did not show the same strong response might be because these calves were older, which would be consistent with a reduction in parental investment in favour of foraging activity (Szabo and Duffus, 2008), or might also be caused by individual variation.

### Risk of hearing effects

The source produced the relatively high received levels at the whale that were needed to invoke behavioural responses; the highest unweighted SEL_cum_ was 183 dB re. 1 µPa^2^ s (Table S1, Fig. S1). Direct measurements of temporary (TTS) or permanent hearing threshold shift (PTS) for humpback whales, or any other baleen whale, do not currently exist. The National Marine Fisheries Service has recently published updated risk thresholds, including an estimate of TTS onset (defined as a TTS of 6 dB) for low-frequency hearing specialist cetaceans, which includes humpback whales, at frequency-weighted SEL_cum_=179 dB re. 1 µPa^2^ s (NMFS, 2016). The NMFS (2016) auditory weighting function for these low-frequency cetaceans is 0 dB at 1.3–2.0 kHz; therefore, the unweighted and weighted SEL_cum_ of the sonar are equal and the SEL_cum_ calculated from the DTAG recordings (Fig. S1) can be directly compared with the risk thresholds of NMFS (2016). This comparison suggests that some of the subject animals may have experienced small amounts of TTS. However, the highest SEL_cum_ is still far (16 dB) below the PTS risk threshold of 199 dB re. 1 µPa^2^ s (NMFS, 2016). Small threshold shifts in hearing sensitivity should have fully recovered after 1 h, and the 2.5–5% duty cycle of the sonar should have resulted in additional recovery of hearing during periods without direct path arrivals of sonar pulses, so it is unlikely that the reduced responsiveness during RampUp2 was the result of TTS from exposures during RampUp1. However, it is worthwhile to note that the reduction in SEL_cum_ achieved by the ramp-up protocol in this study for the responding animals is relevant, as it reduced the risk of TTS in some of those individuals based on these published risk criteria.

### Methodological considerations

We used a sonar source that was previously tested as a prototype sonar system on operational Royal Netherlands Navy frigates. Its maximum SL falls in the low end of the range of operational tactical naval sonars (Ainslie, 2010). By design, ramp-up uses transmissions at reduced SL to decrease the exposure at the animal, and therefore maximum SL was not a crucial factor in determining whether ramp-up is effective. However, theoretical modelling predicted that reductions in received level should be greater for sonar sources with higher maximum SLs, because animals are more likely to respond at greater distances (von Benda-Beckmann et al., 2016). The effectiveness of ramp-up is likely also determined by factors other than SL such as pulse interval, ship speed, whether the source is moving or stationary, and the animals' avoidance behaviour (von Benda-Beckmann et al., 2014, 2016; Wensveen et al., 2015a).

The initial plan was that each experiment would consist of one no-sonar control and two ramp-up sessions during the first season and one no-sonar control, one full-power and one ramp-up session during the second season. Full-power sessions would only be conducted after the first season because of ethical concerns that the sudden onset at maximum SL might cause severe behavioural disturbance. The order of the full-power and ramp-up session would be alternated per new experiment. Based on previous reports of responses of humpback whales to naval sonar (Maybaum, 1989) and other non-impulsive anthropogenic noise (Baker et al., 1983; McCauley et al., 1996), our expectation was that most avoidance responses would occur at SPLs of ∼120–140 dB re. 1 µPa. However, exploratory analyses conducted after the first season indicated that the subject whales were relatively unresponsive. As the full-power protocol was determined to not give whales that avoided the source enough time to significantly affect the risk indicators, the decision was made to use modelled received levels that were based on hypothetical exposures using no-sonar control sessions. This adaptation of the original experimental design greatly increased the statistical power to detect effects. The lack of overt behavioural responses during ramp-up sessions in the first year also alleviated the initial welfare concerns about the full-power sessions. Therefore, we decided *a priori* to conduct two full-power sessions during the second season, to investigate potential effects of the sudden nearby onset of sonar on the magnitude of the behavioural response. While two observations would never have provided conclusive evidence, they could have provided relevant context to our results and indications of an effect of the sudden nearby onset.

### Implications for conservation

Future marine conservation and management efforts critically rely upon our understanding of the acoustic environment as perceived by animals, the spatial and temporal patterns of disturbance, and immediate and chronic responses of animals to disturbance (Chen and Koprowski, 2015). Adaptive management of acoustic disturbance requires evaluation of mitigation methods such as ramp-up or mitigation zones, which require sonar shut-down (Dolman et al., 2009). The heterogeneity in behavioural responsiveness of baleen whales to sonar observed here and in other studies (e.g. Dunlop et al., 2013; Goldbogen et al., 2013) suggests that both between- and within-species variation should be considered when performing environmental risk assessments and evaluation of noise mitigation measures.

Our results represent the first experimental examination of ramp-up for naval sonar. The humpback whale was considered an appropriate model species for this purpose because the whales are relatively easy to find, tag and track. We found that gradually increasing the source intensity was not an effective method to reduce risk of physiological effects for humpback whales overall, because most whales did not exhibit very strong avoidance responses to the sonar signals. This is consistent with recent findings of migrating humpback whales exposed to airgun ramp-up in Australian waters (Dunlop et al., 2016). However, more detailed analyses suggested that ramp-up of sonar reduces risk more effectively in situations in which animals might be more responsive, e.g. when animals have not been exposed recently, animals are in a non-feeding state, or a small calf is present. This suggests that ramp-up will have greater benefits for species that are more behaviourally responsive to sonar than humpback whales. These conclusions should not be extrapolated to ramp-up procedures for other anthropogenic sources, such as seismic airguns, as these sources and their ramp-up procedures are generally very different (Dunlop et al., 2016). Also, when animals have strong motivations not to move away from their current location, ramp-up may not be effective. Therefore, other operational mitigation procedures, e.g. warning sounds prior to sonar pulses (Nachtigall and Supin, 2013), should be considered in addition to ramp-up when mitigation strategies are designed.

The tagged whales were in the line-of-approach of the source ship during experiments and animals may have responded differently if the transmitting source was stationary or moving away. However, a whale directly in front of an approaching sonar ship is most likely to be at risk of physiological damage, and the purpose of ramp-up of sonar is specifically to reduce that risk for whales that happen to be directly in the path of the source ship before it starts a full-level sonar operation.

## APPENDIX A

### Simulation to assess the effect that avoidance could have on SPL_max_ given the full-power protocol

**Fig. A1.**
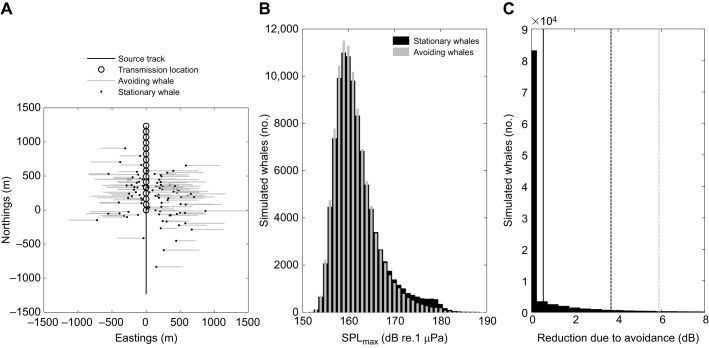
**Simulation to assess the effect that avoidance could have on SPL_max_ given the protocol of the full-power session.** (A) The track of the simulated source, with the source moving in northwards direction, and the locations for a subsample of simulated whales. (B) Monte Carlo distributions of SPL_max_ for all avoiding whales versus all stationary whales. (C) Monte Carlo distribution of the reduction in SPL_max_ caused by simulated avoidance responses, i.e. the difference between the two data sets shown in B. The mean and 95th and 97.5th percentiles are indicated by a dark solid line, dark dashed line and light dashed line, respectively.

A simulation was conducted to validate the assertion that whales could not reduce the risk indicators substantially by moving away in response to sonar during the full-power treatment. Specifically, we estimated the reduction in SPL_max_ that would result from an avoidance response to the first sonar transmission in the full-power session by comparing statistical distributions of SPL_max_ between simulated whales that were stationary throughout the session and simulated whales that started moving in a direction perpendicular to the source track after the first transmission (Fig. A1). A fixed location was assigned to 50% of the simulated whales; the other 50% initiated avoidance and kept moving until sonar transmissions ceased. In each realization of the simulation, a simulated whale was assigned a different horizontal speed, depth and starting position. These values were randomly drawn from distributions fitted to actual whale positions observed at *t*=5 min during no-sonar control and full-power sessions, and horizontal speed and depth of the whales during the observed avoidance responses. For whale starting positions, distance to the source was drawn from a truncated normal distribution with mean µ=456 m, s.d σ=185 m and lower bound=0 m, and the angle to the source was drawn from a von Mises distribution with µ=20 deg and concentration κ=0.89. Whale depth was drawn from a gamma distribution with shape *a*=0.5 and scale *b*=37 m, and horizontal avoidance speed was drawn from a truncated normal distribution with µ=1.3 m s^−1^, σ=0.5 m s^−1^ and lower bound=0 m s^−1^. The simulated source followed the navigational and transmission protocol of the full-power session (Fig. A1); horizontal speed=4.1 m s^−1^, depth=50 m, and one pulse was transmitted at full power every 20 s between *t*=5 and *t*=10 min. We estimated SPL_max_ by assuming spherical spreading without absorption. The simulation only considered SPL_max_ because SEL_cum_ depends strongly upon SPL_max_ for close ship approaches. Simulations were run with 100,000 iterations.

The 75th, 95th and 97.5th percentiles of the reduction in SPL_max_ owing to avoidance were 0, 3.7 and 5.9 dB, respectively (Fig. A1). This indicated that there was a negligible chance during full-power sessions that a whale could substantially reduce its SPL_max_ by avoiding, given the experimental protocol of the full-power session and typical observed intercept distance, depth and avoidance speed of the whales.

One reason for this result was likely the large difference in speed between the source (4.1 m s^−1^) and the receiver (∼1.3 m s^−1^). All simulated whales avoided instantaneously and in a direction perpendicular to the source track (Fig. A1), which should maximize the reduction in received level (Wensveen et al., 2015a). However, actual observed avoidance responses had no instantaneous turn and generally less ‘optimal’ headings (often 0 deg relative to the course of the source; Fig. 2C, Fig. S2). Simulations were informed by parameters derived from avoidance responses during ramp-up sessions, and it is possible that the full-power protocol would, on average, induce stronger responses that would reduce risk of hearing loss more effectively. However, no avoidance responses were observed in the experimental two full-power sessions conducted, but both these whales were previously exposed.

## APPENDIX B

### Details of the received level analysis

We modelled acoustic PL using ray-trace software (BELLHOP; Porter and Bucker, 1987) to obtain the received levels for all sonar sessions, i.e. ramp-up sessions and simulated and observed full-power sessions. The acoustic model assumed a pressure-release sea surface and a bottom layer that was a flat, homogeneous fluid layer with constant acoustic properties. Bottom reflection coefficients were calculated using geo-acoustic parameters reported in Ainslie (2010) for the most prevalent sediment types at the exposure sites (Gurevich, 1995), in combination with the water sound speed and density, derived from CTD data, above the seafloor. For each experimental session, incoherent PL was modelled for a single two-dimensional slice with 1×1 m grid resolution. Each slice was 4 km long, and its vertical dimension was taken as the mean sea floor depth, based on BODC (2010), between the source and whale at the start of the experimental session.

The modelled sound source was based on the properties of the real sonar source. We modelled PL at 1.6 kHz, i.e. the logarithmic middle of the band. The vertical directivity pattern of the real source at that frequency (3 dB beamwidth: ±40 deg) was implemented. The range of beam take-off angles in the vertical plane was ±89 deg. The number of traced Gaussian beams was 3200 (this number was automatically selected by BELLHOP). The modelled source was horizontally omnidirectional and was placed at the mean tow depth of the real source calculated over all pulses in the experimental session.

The energy source level (SL_E_) was derived from the SL based on mean-square sound pressure via SL_E_=SL+10log_10_(*T*/*t*_0_) dB, where *t*_0_ is 1 s. Because of a gradual onset and offset in the waveform, the effective duration *T* of a transmitted sonar pulse was 0.47 s and 0.93 s for the time intervals 0≤*t*<5 min and 5≤*t*≤10 min, respectively. The received single-pulse SEL and single-pulse SPL were derived from the modelled PL as SEL=SL_E_−PL and SPL=SL−PL, respectively. SEL_cum_ of the experimental session was then calculated by taking 10 times the 10-base log of the sum of the single-pulse sound exposures *E* received in the session, where *E*=10^SEL/10 dB^ µPa^2^ s. SPL_max_ was taken as the maximum over all SPL in the session.

We applied a Monte Carlo method to propagate forward uncertainty in the three-dimensional position of the whale into the received levels (SPL_max_ and SEL_cum_). For whale depth we used a normal distribution centered at the depth measured by the DTAG at the time of the sonar transmission and s.d.=1 m reflecting the precision of the pressure sensor (Johnson and Tyack, 2003). Wensveen et al. (2015b) used a Bayesian method to reconstruct probabilistic horizontal tracks for the humpback whales in this study from the DTAG data (orientation, depth, flow noise), visual observations of range and bearing to the whale, and Fastloc-GPS positions. Therefore, we derived the uncertainty in horizontal source–whale range from the uncertainty in the spatial location of the whale track. Depending on whale and session, the s.d. for SPL_max_ ranged from 0 to 1.2 dB and the s.d. for SEL_cum_ ranged from 0 to 0.8 dB. Uncertainty in the horizontal track of the whale and, therefore, uncertainty in received SPL_max_ and received SEL_cum_ were larger for data sets without Fastloc-GPS positions (Wensveen et al., 2015b).

For all humpback whales exposed to sonar, Sivle et al. (2015) described how the received levels of the pulses were calculated from the acoustic recordings made by the DTAGs. Received levels were calculated using tag-specific mean sensitivity values based on calibration measurements conducted in the 1–2 months before each research trial in 2011, 2012 and 2013 (for more details, see Wensveen, 2016). On average, the modelled SPL_max_ was 5.8 dB lower than the SPL_max_ measured in the DTAG recordings, and this difference was 2.2 dB for SEL_cum_ (Fig. S1). This mean difference based on all sessions measured was added to the modelled received level, reflecting our higher confidence in the accuracy of the measurements. This correction only affected absolute values and did not affect the main results of this study, as they are based on differences in received level. Potential reasons for the discrepancy between the modelled and measured levels included the simplified assumptions of the acoustic modelling, limited environmental information and the difference in root mean square averaging time for the SPL calculations. In addition, the total SL_E_ calculated over all pulses in the ramp-up session was 0.43 dB higher than the total SL_E_ calculated over all pulses in the full-power session (Fig. 1). Therefore, we subtracted 0.43 dB from the SEL_cum_ for ramp-up sessions so that any potential statistical differences between RampUp and FullPower in this metric were caused by differences in behavior and not by differences in transmission protocols.

## Supplementary Material

Supplementary information
