# Supplementary material for: Lack of behavioural responses of humpback whales (Megaptera novaeangliae) indicate limited effectiveness of sonar mitigation
Source: J Exp Biol. 2017 Nov 15;220(22):4150–61. doi: 10.1242/jeb.161232 (PMC5702040; doi:10.1242/jeb.161232)
Supplement: Supplementary information [file jexbio-220-161232-s1.pdf]

Table S1. Data table with information used in the statistical analysis.

Tag	Whale	Group	SPLmax	SEL	Rmin	Session	Order	Avoidance	Feed
mn11_157a	1	1	170.39	172.30	350.8	0	0	0	0
mn11_157a	1	1	162.25	169.26	998.0	1	0	1	0
mn11_157a	1	1	179.88	181.35	62.3	2	1	0	0
mn11_158a	2	2	173.64	177.91	221.2	0	0	0	1
mn11_160a	3	3	169.55	175.62	368.7	0	0	0	0
mn11_160a	3	3	177.37	179.29	128.6	1	0	0	0
mn11_160a	3	3	168.88	173.87	417.3	2	1	0	0
mn11_165e	4	4	180.47	182.75	80.3	0	0	0	1
mn11_165e	4	4	176.57	179.30	161.1	1	0	0	1
mn11_165e	4	4	175.00	180.96	166.2	2	1	1	0
mn12_161a	6	5	165.68	172.56	858.3	1	0	1	0
mn12_161a	6	5	168.42	171.82	501.9	0	1	0	0
mn12_164a	7	6	174.72	175.82	154.0	1	0	1	1
mn12_164a	7	6	177.04	180.02	61.0	0	1	0	1
mn12_170a	8	7	178.68	179.36	93.0	0	0	0	0
mn12_170a	8	7	173.24	177.58	248.9	1	0	0	1
mn12_170a	8	7	171.86	177.65	411.4	2	1	1	1
mn12_170b	9	7	178.70	179.32	108.5	0	0	0	0
mn12_170b	9	7	173.72	177.77	305.7	1	0	0	1
mn12_170b	9	7	172.41	177.96	468.8	2	1	1	1
mn12_171b	10	8	177.55	181.79	136.4	0	0	0	1
mn12_171b	10	8	174.74	177.33	207.6	1	0	0	1
mn12_171b	10	8	178.09	180.16	67.2	2	1	0	1
mn12_178a	11	9	175.15	179.17	190.7	0	0	0	1
mn12_178a	11	9	167.72	174.28	499.3	1	0	0	0
mn12_178a	11	9	172.23	177.04	231.6	2	1	0	1
mn12_179a	12	10	175.55	179.61	202.8	0	0	0	0
mn12_179a	12	10	171.75	177.56	347.8	1	0	1	0
mn12_179a	12	10	173.93	178.71	236.6	2	1	0	0
mn12_180b	13	11	171.81	177.40	366.2	0	0	0	1
mn12_180b	13	11	171.68	175.95	400.3	1	0	1	1
mn12_180b	13	11	172.10	176.61	320.5	2	1	1	0

Journal of Experimental Biology • Supplementary information

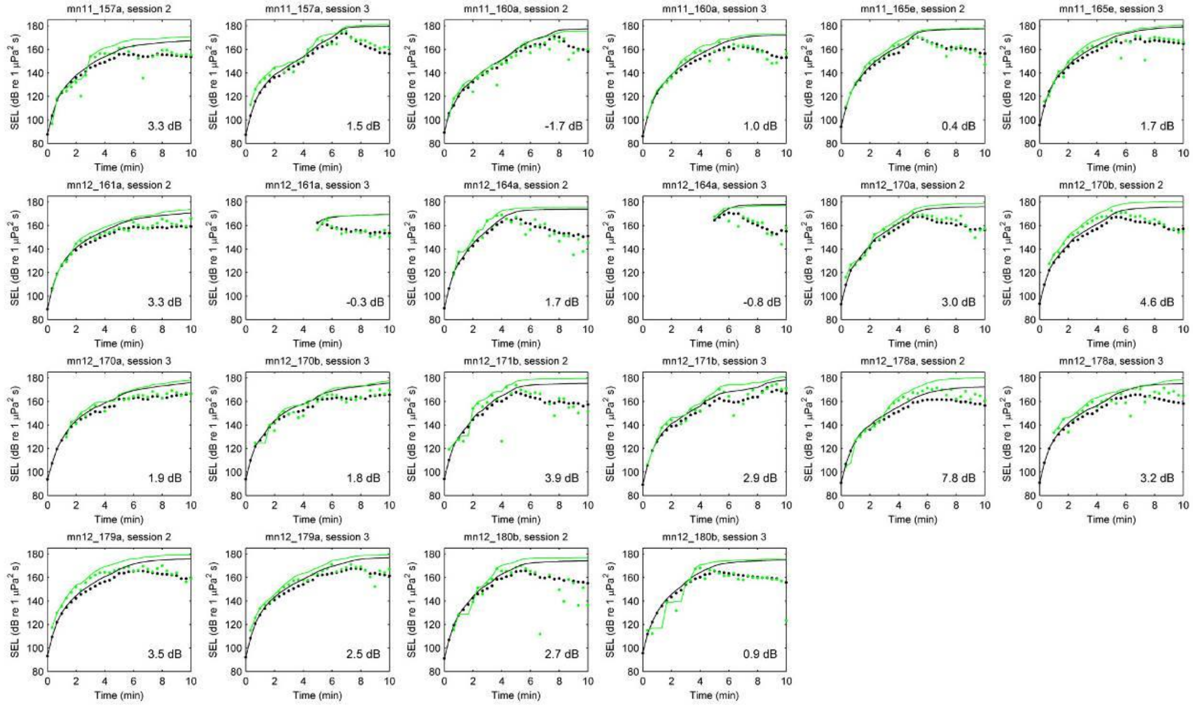

Fig. S1. Received sound exposure levels (SELs) of all pulses transmitted during sonar sessions. The measured levels were calculated from the acoustic recordings made with the DTAG (green) and modelled levels were based on propagation loss predictions made using BELLHOP (black). Single-pulse SEL is shown with a dot and SEL accumulated over all pulses in the experimental session is shown with a line. The value in the bottom right corner of each panel represents the difference in cumulative SEL at the end of the session, i.e. measured SEL minus modelled SEL, before corrections were applied.

Journal of Experimental Biology • Supplementary information

Fig. S2. Horizontal tracks, heading data and dive profiles for all humpback whales. Experiment IDs: A) mn11_157, B) mn11_158, C) mn11_160, D) mn11_165, E) mn12_161, F) mn12_164, G) mn12_170, H) mn12_171, I) mn12_178, J) mn12_179, and K) mn12_180. Maps of the area where vessel approaches with humpback whales were conducted: no-sonar control session (top left), first sonar session (top middle; RampUp1), and second sonar session (top right; RampUp2 or FullPower). The horizontal track of the whale (line) is shown in black with the section corresponding to the experimental session highlighted in red. The location of the sonar source (grey dots), and the location of the research vessel towing the source (grey circles) are shown for each sonar transmission. Timeseries data for the same experimental sessions are shown below the maps, with the following variables plotted as function of time relative to the start of the session: 1) whale heading relative to North, 2) whale heading relative to the course of the ship (where 0º represents whale movement in the same direction and 180º in the opposite direction), and 3) depth of the whale overlaid with circles indicating when lunge feeding events occurred. The onset of avoidance during sessions is marked in the timeseries plots with a red vertical line. Data available for the non-focal whale (mn11_165f, mn12_170a) are shown in grey for experiments in which two whales were tagged. Note the difference in depth scales between figures.

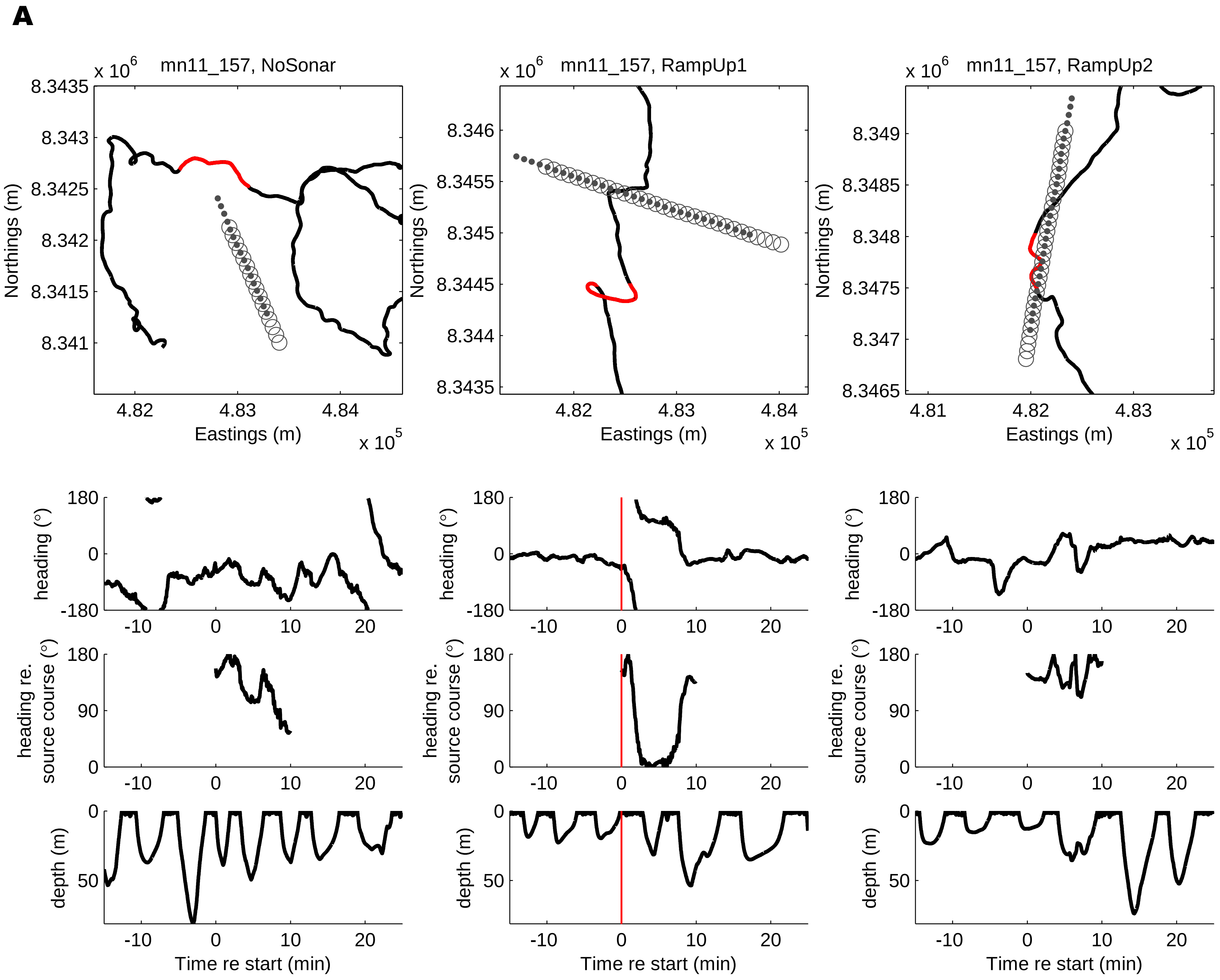
A
mn11_157, NoSonar
mn11_157, RampUp1
mn11_157, RampUp2
Northings (m)
Eastings (m)
x 10^6
x 10^5
heading (°)
heading re. source course (°)
depth (m)
Time re start (min)

B

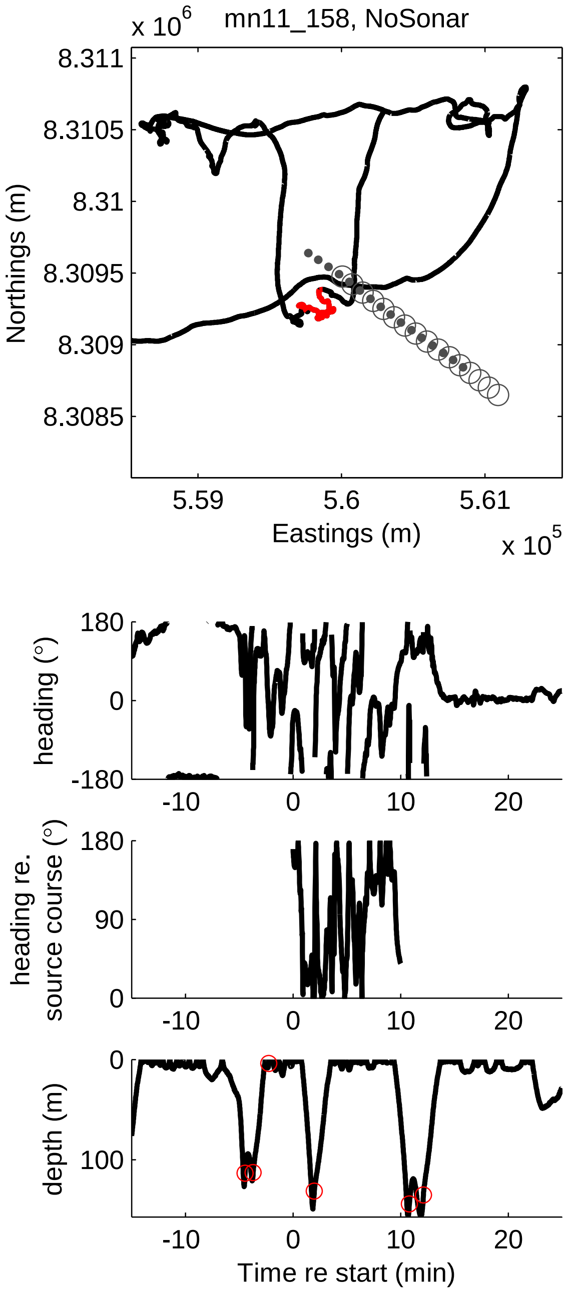

Journal of Experimental Biology • Supplementary information

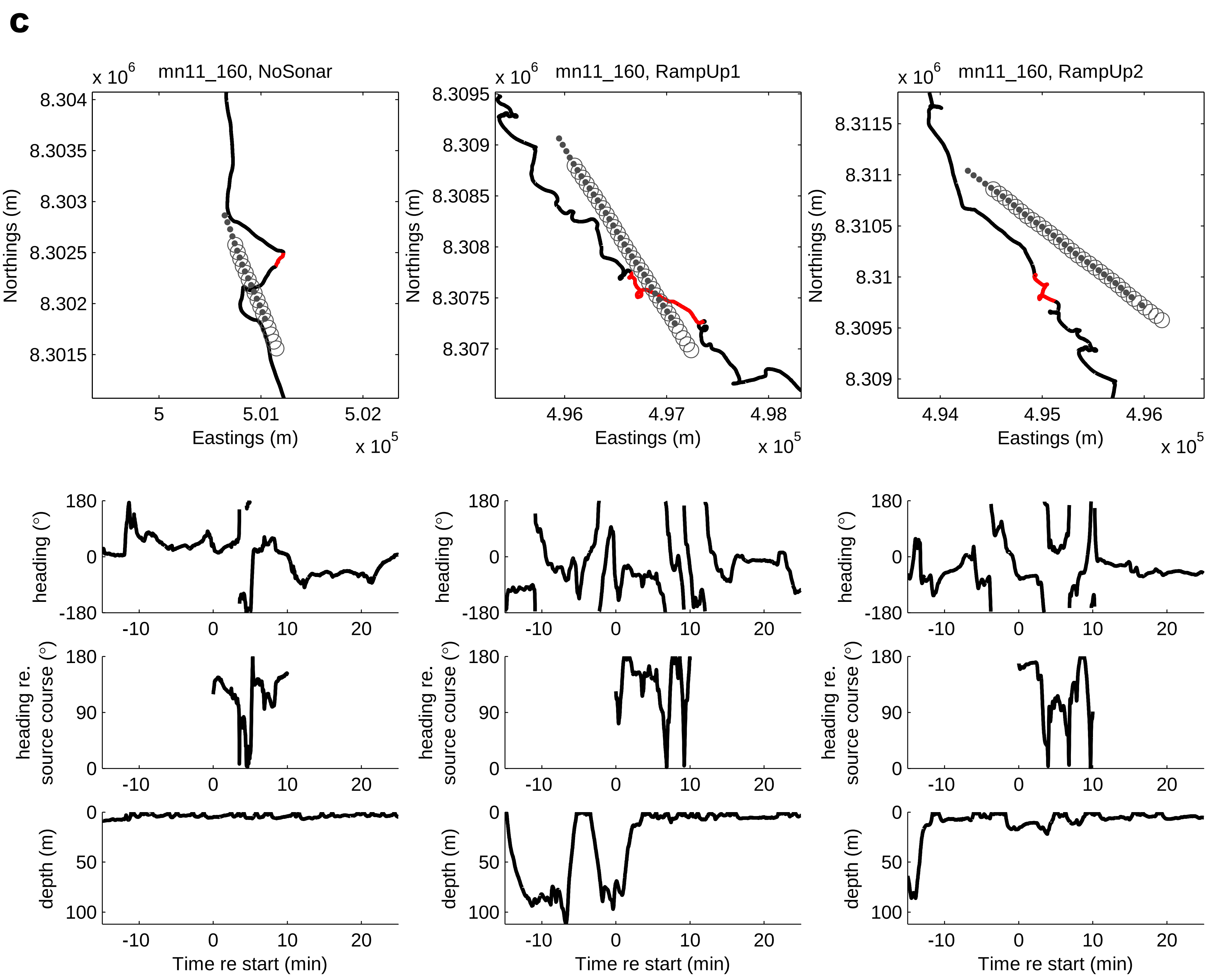

Journal of Experimental Biology • Supplementary information

D

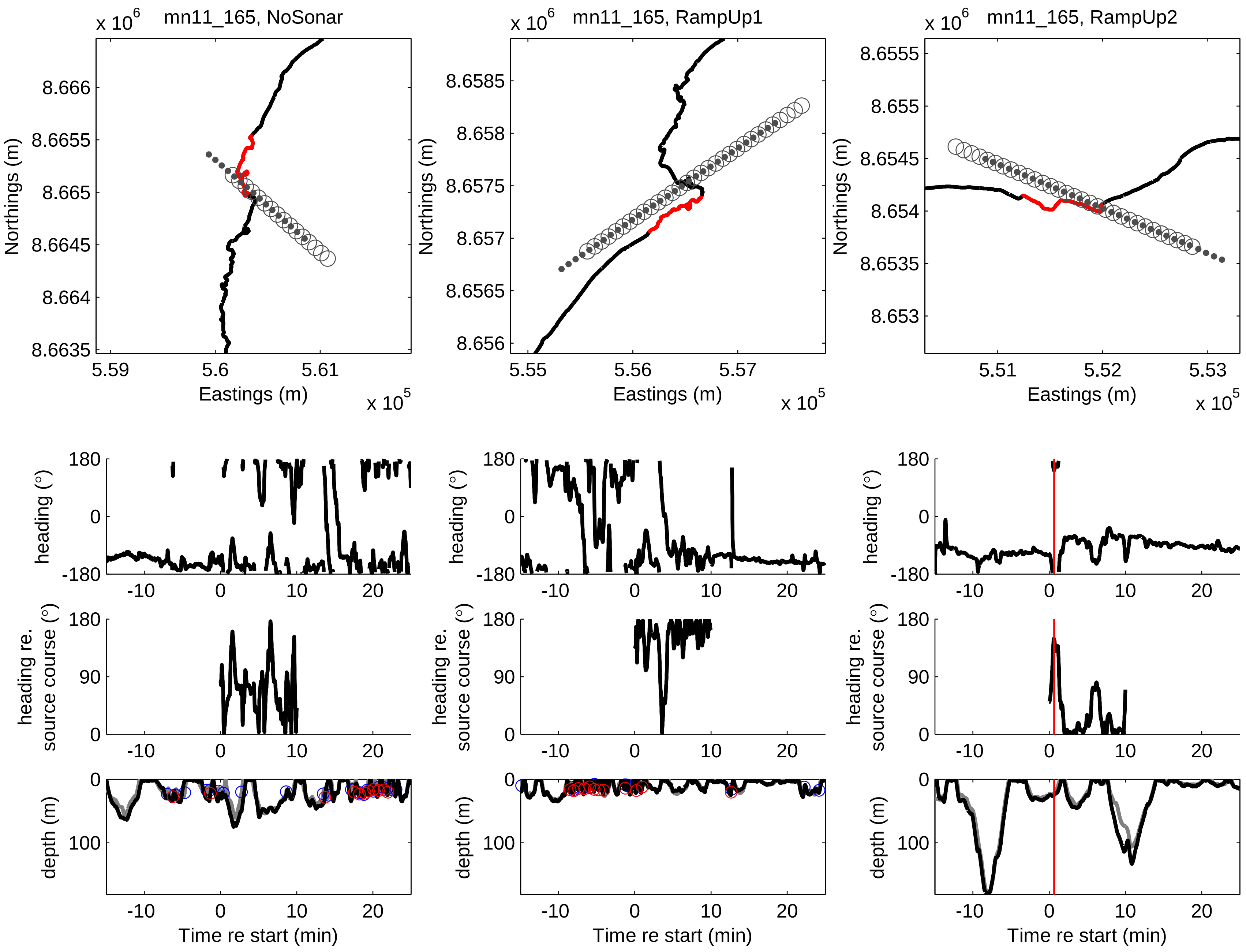

Journal of Experimental Biology • Supplementary information

E

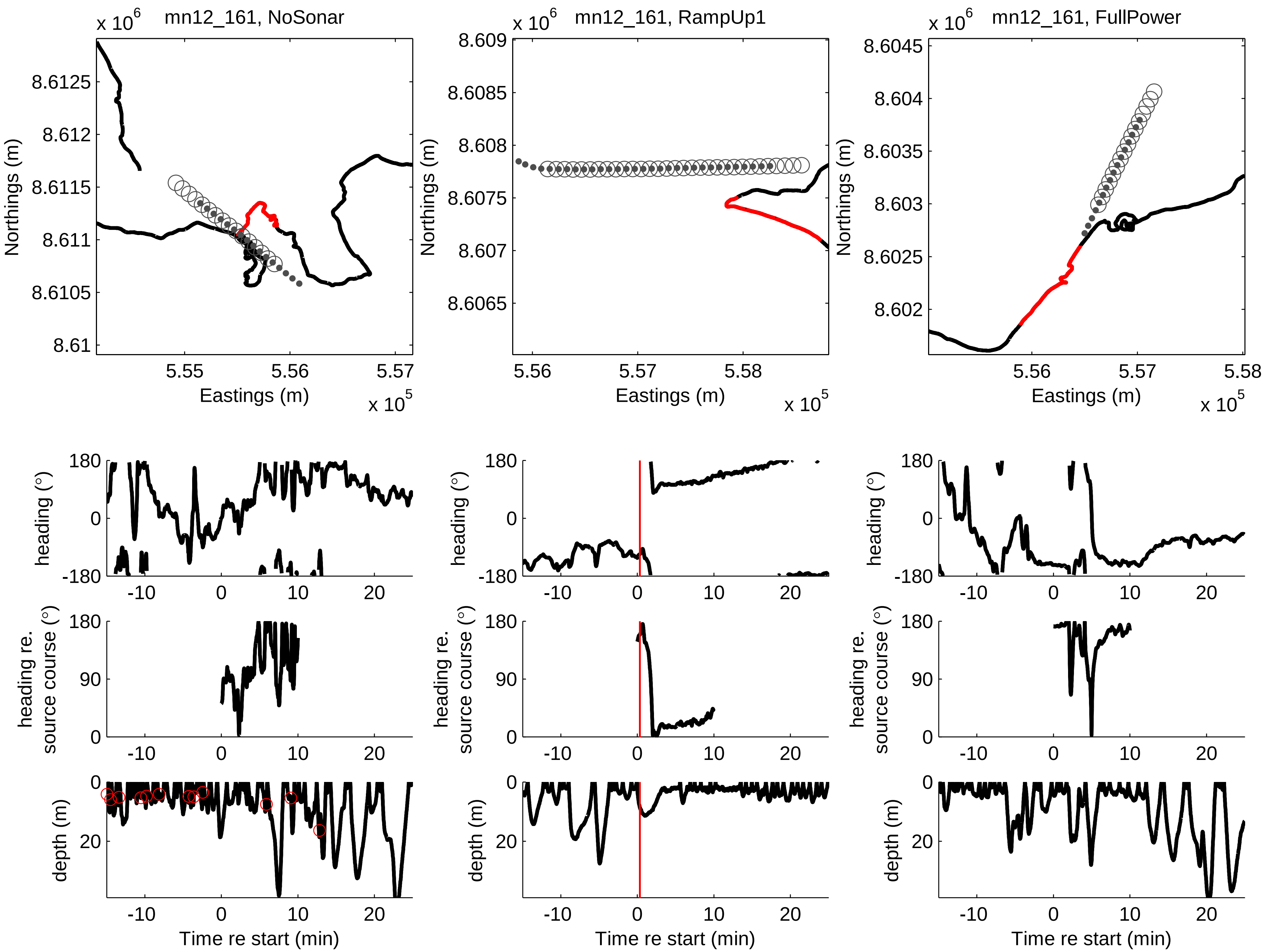
mn12_161, NoSonar
mn12_161, RampUp1
mn12_161, FullPower
Northings (m)
Eastings (m)
x 10^6
x 10^5
heading (°)
heading re. source course (°)
depth (m)
Time re start (min)

F

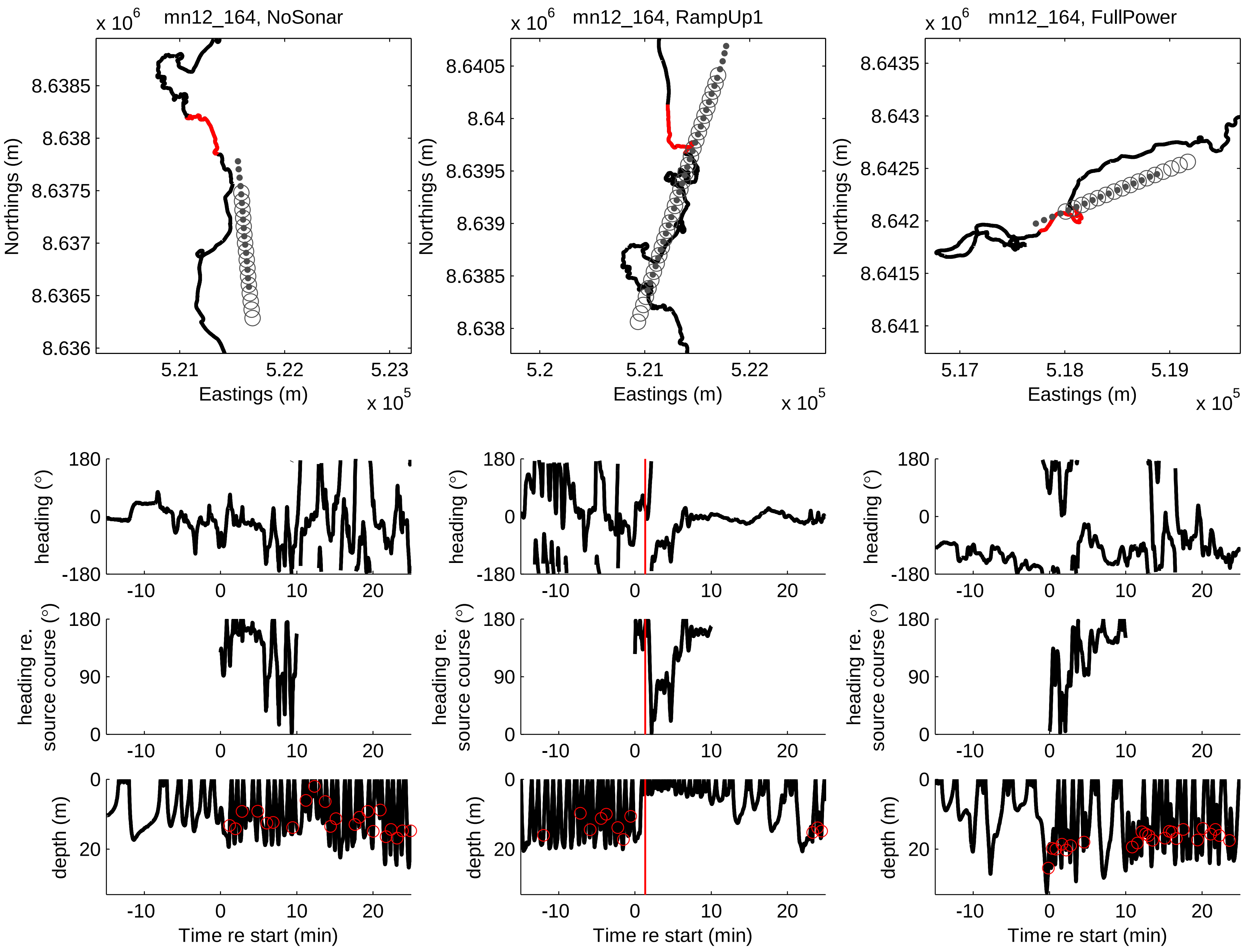

mn12_164, NoSonar
mn12_164, RampUp1
mn12_164, FullPower
Northings (m)
x 10^6
Eastings (m)
x 10^5
heading (°)
heading re. source course (°)
depth (m)
Time re start (min)

G

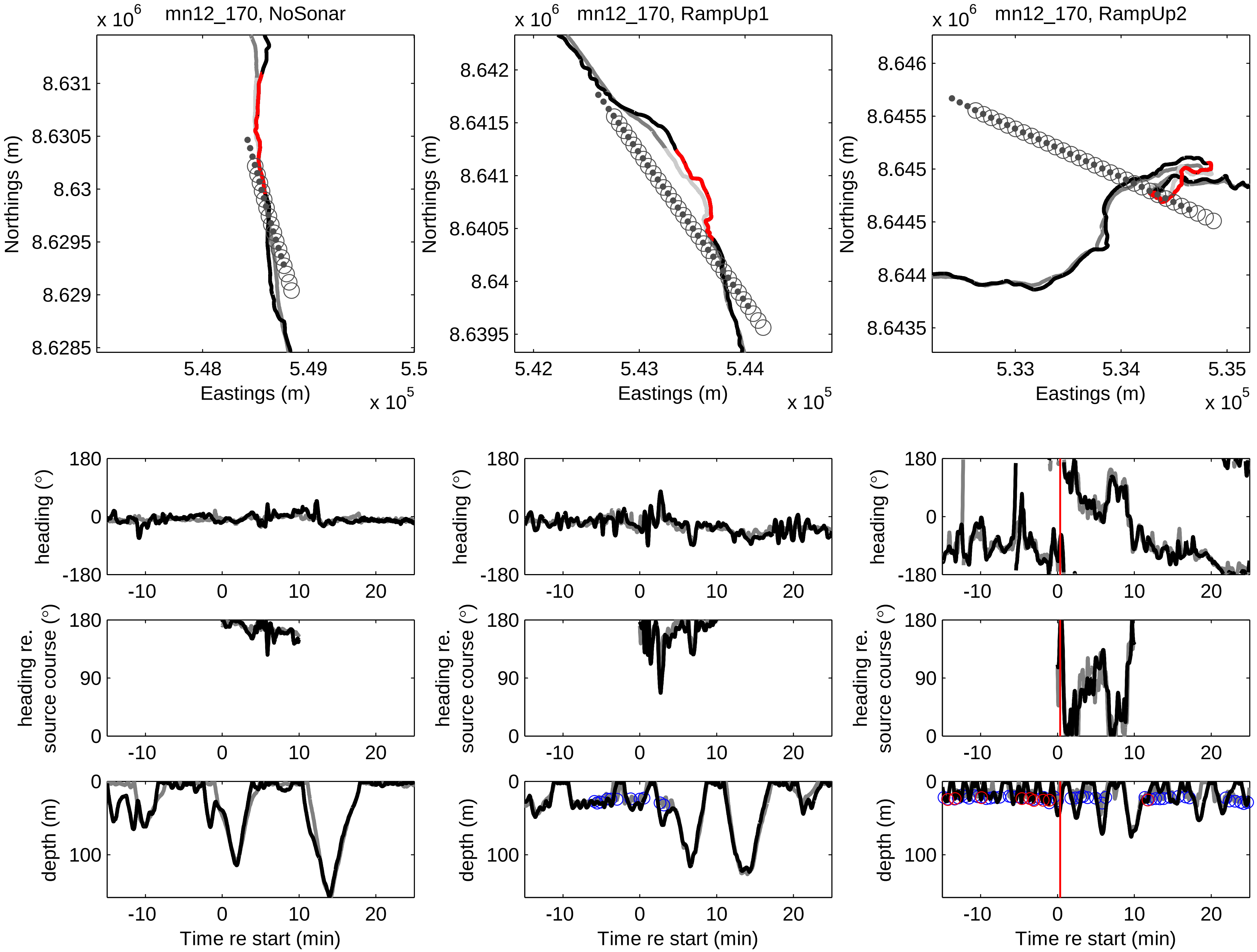

Journal of Experimental Biology • Supplementary information

H

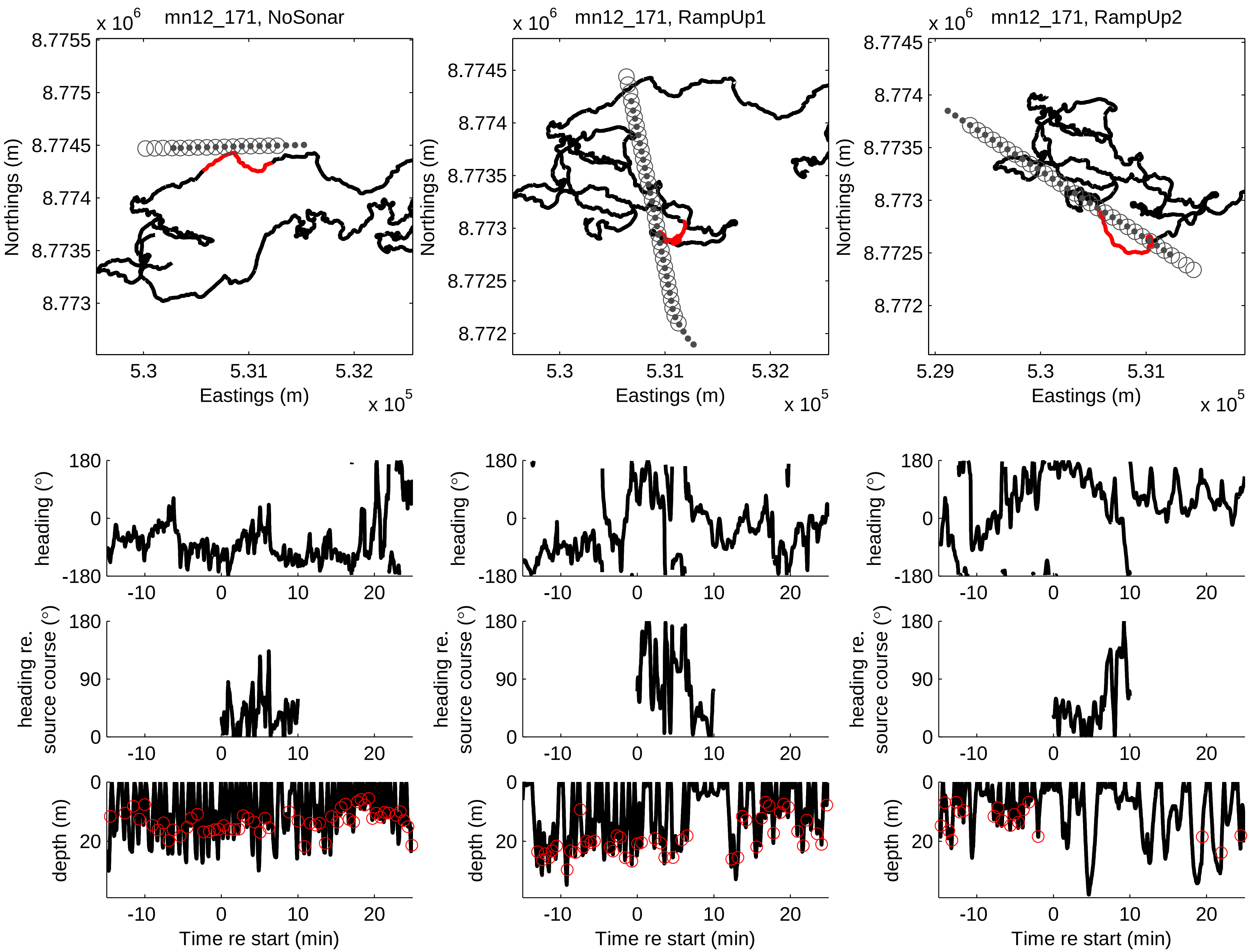

Journal of Experimental Biology • Supplementary information

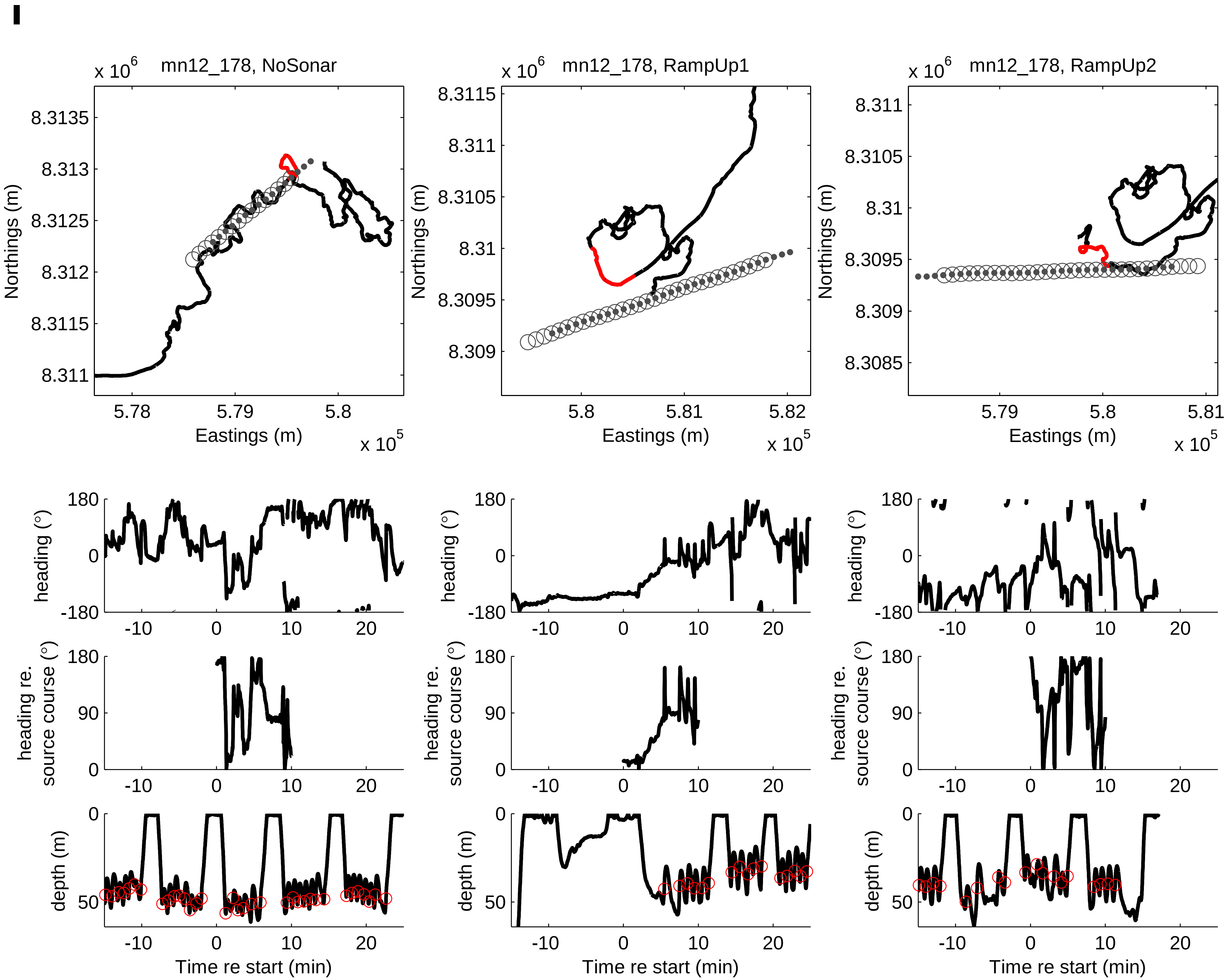

Journal of Experimental Biology • Supplementary information

J

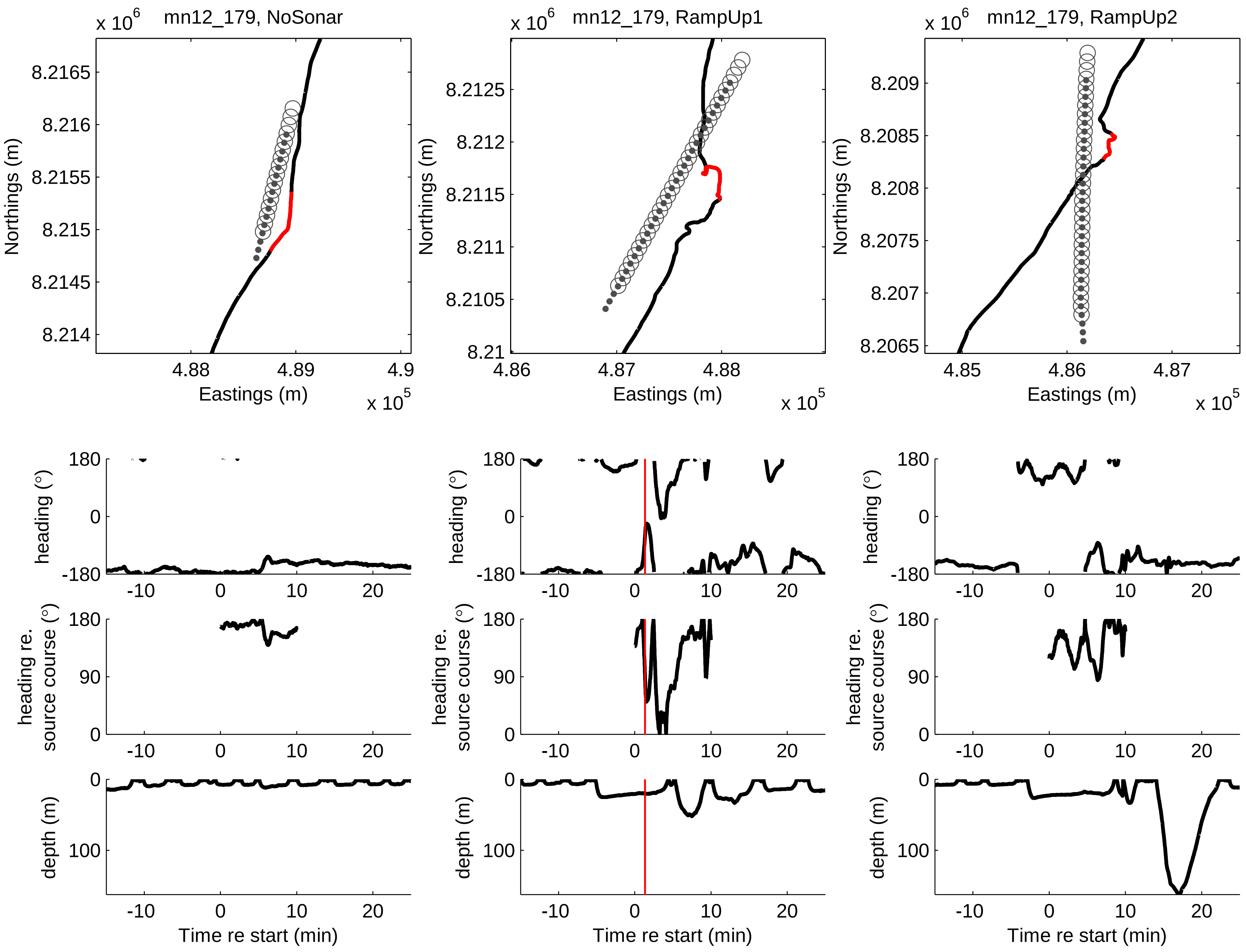

Journal of Experimental Biology • Supplementary information

K

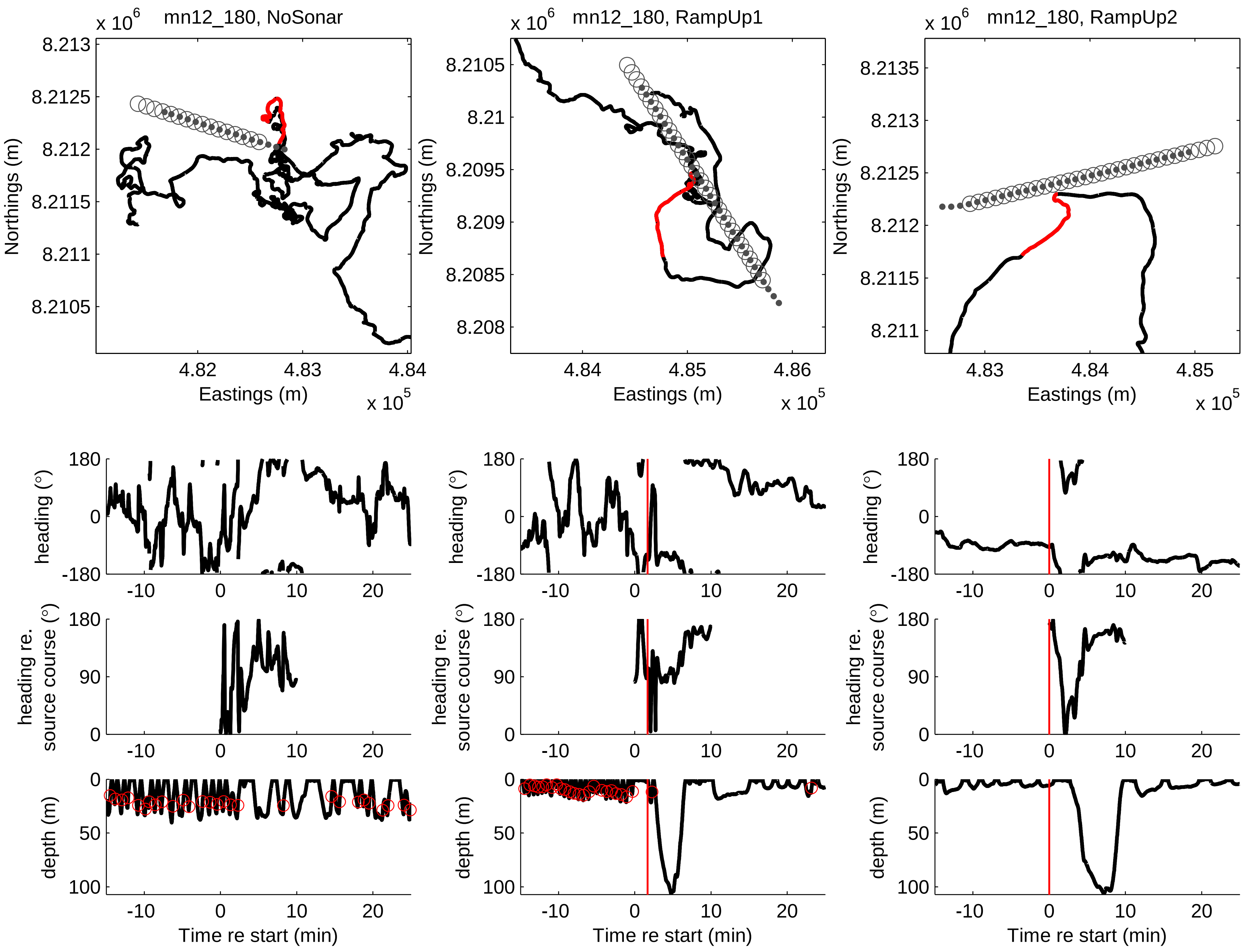

Journal of Experimental Biology • Supplementary information
